# Chromosome–nuclear envelope attachments affect interphase chromosome territories and entanglement

**DOI:** 10.1186/s13072-018-0173-5

**Published:** 2018-01-22

**Authors:** Nicholas Allen Kinney, Igor V. Sharakhov, Alexey V. Onufriev

**Affiliations:** 1 Genomics Bioinformatics and Computational Biology, Virginia Tech, Blacksburg, VA 24061 USA; 2 Department of Entomology, Virginia Tech, Blacksburg, VA 24061 USA; 3 Laboratory of Ecology, Genetics and Environmental Protection, Tomsk State University, Tomsk, Russia 634050; 4 Department of Physics, Virginia Tech, Blacksburg, VA 24060 USA; 5 Department of Computer Science, Virginia Tech, Blacksburg, VA 24061 USA; 6 Center for Soft Matter and Biological Physics, Virginia Tech, Blacksburg, VA 24061 USA

**Keywords:** *Drosophila melanogaster*, Chromatin, Nuclear envelope, Chromosome territories

## Abstract

**Background:**

It is well recognized that the interphase chromatin of higher eukaryotes folds into non-random configurations forming territories within the nucleus. Chromosome territories have biologically significant properties, and understanding how these properties change with time during lifetime of the cell is important. Chromosome–nuclear envelope (Chr–NE) interactions play a role in epigenetic regulation of DNA replication, repair, and transcription. However, their role in maintaining chromosome territories remains unclear.

**Results:**

We use coarse-grained molecular dynamics simulations to study the effects of Chr–NE interactions on the dynamics of chromosomes within a model of the *Drosophila melanogaster* regular (non-polytene) interphase nucleus, on timescales comparable to the duration of interphase. The model simulates the dynamics of chromosomes bounded by the NE. Initially, the chromosomes in the model are prearranged in fractal-like configurations with physical parameters such as nucleus size and chromosome persistence length taken directly from experiment. Time evolution of several key observables that characterize the chromosomes is quantified during each simulation: chromosome territories, chromosome entanglement, compactness, and presence of the Rabl (polarized) chromosome arrangement. We find that Chr–NE interactions help maintain chromosome territories by slowing down and limiting, but not eliminating, chromosome entanglement on biologically relevant timescales. At the same time, Chr–NE interactions have little effect on the Rabl chromosome arrangement as well as on how chromosome compactness changes with time. These results are rationalized by simple dimensionality arguments, robust to model details. All results are robust to the simulated activity of topoisomerase, which may be present in the interphase cell nucleus.

**Conclusions:**

Our study demonstrates that Chr–NE attachments may help maintain chromosome territories, while slowing down and limiting chromosome entanglement on biologically relevant timescales. However, Chr–NE attachments have little effect on chromosome compactness or the Rabl chromosome arrangement.

**Electronic supplementary material:**

The online version of this article (10.1186/s13072-018-0173-5) contains supplementary material, which is available to authorized users.

## Background

The three-dimensional (3D) organization of the genome (chromatin) plays an important role in key cellular processes such as DNA replication, repair, transcription [1], and epigenetic inheritance [2]. Links between chromatin architecture and diseases such as cancer are being established [3]. Unlike most proteins that adopt the same unique 3D shapes in all cells, the conformational states of the chromatin fiber are not nearly as compact or ordered and are stochastic to some degree. Remarkably, several features of chromatin folding appear to be universal. Chromosomal territories, in which each chromosome occupies a distinct region of the nucleus, have been observed in numerous organisms and cell types, such as yeast [4], human [5], *D. melanogaster* (fruit fly) [6–8], mouse [9], and *Arabidopsis* [10]. Chromosome interactions, both within (intra) chromosomes and between (inter) chromosomes, have been observed microscopically [6, 8] and inferred using cross-linking techniques [11] such as the Hi-C method. Intra-chromosomal interactions in particular are often characterized by their power law decay which may differ among organisms [11, 12]. Chromosomal entanglement, characterized by knots which hamper chromosome folding and unfolding, appears to occur infrequently based on direct observations in *D. melanogaster* [8] and both experimental and computational studies in human [11, 13]. Chromosomes in yeast [4], fruit fly [6–8], and *Arabidopsis* [10] possess a distinctly polarized (Rabl) chromosome arrangement characterized by a separation of chromosome centromeres and telomeres; the arrangement is thought to be a remnant of anaphase.

One way to quantify the compactness of a chromosome is by measuring, e.g., using Hi-C, the probability P that two loci on the same chromosome polymer are in contact with each other in 3D space. This probability can be related to the genomic distance s between these loci along the chromosome: *P*(*s*) = *s*^*α*^. Here, the scaling exponent *α* quantifies the degree of compactness of the chromosomes: smaller values of *α* indicate less compact chromatin. Computational approaches are now routinely used to predict genome-wide folding based on the collection of features revealed by a given experiment. For example, close integration of computation and experiment has been used to suggest that the human genome folds into a shape called the fractal globule (FG) [11, 13]. This shape correctly predicts three key features of experiment: the presence of chromosome territories, lack of chromosome entanglement, and the scaling law *P*(*s*) = s^−1^ [13]. Indeed, the − 1 exponent is a key feature of the fractal globule. Experimentally, the fractal nature of chromatin in eukaryotes was first observed in neutron scattering studies [14], and later by Hi-C technique [11]. Interestingly, the FG is a non-equilibrium state which may imply that the true chromosome configurations suggested by experimental Hi-C maps are also out of equilibrium. Nevertheless, computational approaches have used both equilibrium and non-equilibrium approaches to generate fractal-like chromosome configurations. Equilibrium approaches have used pseudo-Boltzmann distributions to simulate the non-equilibrium properties of fractal configurations [13]. In a recent study, the chromosome configurations generated by this thermodynamic-based approach reiterated many properties of the fractal globule. It was demonstrated that ideal chromosome configurations are largely free of knots and tend to form fibrils reminiscent of the crumples that recursively form the FG [13]. On the other hand, true (fully) equilibrium-based folding models have been rejected due to the absence of chromosome territories, high degree of knotting, and the scaling law *P*(*s*) = *s*^−3/2^ which deviates from Hi-C experimental data [11]. The equilibrium globule (*α* = − 3/2) is less compact than the fractal globule (*α* = − 1): All else being the same, two loci separated by the same distance along the genome are less likely to be in contact in the equilibrium globule.

In *D. melanogaster*, both fractal and hierarchical (modular) shapes have been proposed to explain the folding of interphase chromatin [12]. In the hierarchical model, multiple genes cluster into domains bounded by epigenetic markers positioned along the chromosome fiber. These domains in turn form their own clusters: Inactive domains tend to aggregate, while the less compact active domains tend to facilitate inter-chromosomal interactions [12]. In contrast to the FG, hierarchical chromatin folding is more compartmentalized, reflecting the known epigenetic profiles of the chromosomes; however, both recapitulate the overall pattern of chromosome interactions revealed by Hi-C. A recent study has introduced two additional models: the “tension globule” model and the chromatin extrusion model [15]. The tension globule is formed during polymer condensation by inter-monomer attraction forces [15]. The chromatin extrusion model proposes that CCCTC-binding factor (CTCF) and cohesin partitions unknotted loops of chromatin in a manner consistent with experiment [15]. Each of these models possesses characteristics of the FG while being distinct from it [15]. Thus, chromatin folding predicted in most recent studies [15, 16] has been likened to the theoretical fractal globule [11, 13] with the qualification that chromatin folding may not be strictly fractal [15].

Although most studies now agree that chromosomes in higher eukaryotes fold into a non-equilibrium state which inevitably transitions to equilibrium, there is no consensus at present on the timescales necessary to reach equilibrium. In human, it has been suggested that the FG is a long-lived state and transition to equilibrium is simply longer than the lifetime of the cell [17]. However, other recent studies argue that fractal-like configurations exist along a spectrum connecting open chromatin at one extreme to compact chromatin at the other [18]. In the strings and binders switch model (SBS) [18], this spectrum of configurations is explored by altering the affinity and concentration of binder molecules that mimic the cell’s DNA-binding machinery. The SBS model predicts that fractal-like configurations occurring during the transition from open to compact chromatin states may be fleeting in the presence of topoisomerases [18]. Thus, the duration and stability of fractal-like configurations and chromosome territories remain largely unknown. Statistical models—that generate snapshots of configurational ensembles—have succeeded in reconstructing the folding of *D. melanogaster* chromosomes through the integration of experimental data on chromosome–chromosome and Chr–NE interactions [19]; however, these models are unable to predict the dynamics of chromosomes in interphase.

Here, we investigate the duration and stability of fractal-like configurations in the context of Chr–NE interactions. We use a model of the 3D genome organization in the interphase nucleus of *D. melanogaster* embryonic-derived Kc cells, which have been used for studying the organization and function of the eukaryotic genome [20–26].

Chr–NE interactions are taken from DamID experiments that identified at least 412 lamin-associated domains (LADs), which maintain close proximity to the NE in *D. melanogaster* Kc cells in vivo [25, 26]. The DamID approach is a method based on detecting DNA methylation by a chimeric protein consisting of a chromatin protein fused with methyltransferase [27]. The LAD sites in Kc cells also correlate with sites of chromosome–nuclear envelope (Chr–NE) attachment in polytene chromosomes [25]: the correspondence has important implications in our model. Since NE attachments in polytene chromosomes are known to affect their folding [28, 29], we speculated that Chr–NE attachments may play a similar role in non-polytene chromosomes. However, little is known about the *specifics* of this hypothesis. For instance, could the presence of Chr–NE attachments prolong compact fractal-like configurations, which, given sufficient time, will transition to less compact equilibrium conformations? Are Chr–NE attachments necessary to maintain the Rabl configuration of chromosomes, which is estimated to last over 2 hours in the interphase nucleus of *D. melanogaster* [30]? Chr–NE attachments in the polytene nucleus are known to reinforce chromosome territories and mitigate chromosome entanglement [28, 29]; is this also the case in regular non-polytene interphase chromosomes? Our study aims to answer these questions using a computational model of the *Drosophila* interphase nucleus.

As a model organism, *D. melanogaster* has several critical advantages over others. First, the chromosome interactions with the NE have been comprehensively mapped in DamID experiments [25, 26]. So far, the full complement of these interactions has been mapped for a limited number of organisms. Experimental mapping of these interactions in humans has revealed their clustering into more than 1300 well-defined LADs [31]. Just like in fruit fly, clustered regions of Chr–NE interaction are generally transcriptionally inactive. Having access to the experimentally determined Chr–NE interactions enables the mapping of each interaction onto a computational model of chromosomes in interphase. Second, since the dynamics of specific chromosomal loci are known from experiment [32], we are also able to directly validate the timescale of chromatin dynamics seen in simulations. Third, the initial configuration of chromosomes in the model can be designed to match the fractal-like configurations suggested by experiment [11]. Using these data, this work considers two main models of the *D. melanogaster* interphase nucleus. A *wild-type model* possesses all known parameters of *D. melanogaster* nucleus including the experimentally identified Chr–NE attachments in [25]; a *control (Null) model* is identical to the wild-type model but lacks specific sites of attachment between chromosomes and the NE. The effects of Chr–NE attachments are studied by comparing the dynamics of the wild-type and control models. To ascertain robustness of our conclusions to model assumptions, we have also tested a companion model with Chr–NE attachments mapped from more recent experimental data found in Ref. [26], including a more realistic computational modeling of Chr–NE attachments. Finally, in real cells, the presence of topoisomerases helps relieve torsional stresses and chromosome entanglements. While a full investigation of topoisomerase’s influence on chromosome characteristics is outside of scope of this focused study, here we have demonstrated how a relatively simple approach can be used to investigate these effects computationally. In the context of the present work, we have used the approach to investigate how our conclusions about Chr–NE attachments might be affected by the presence of topoisomerase II.

## Results

### The complex diffusive motion of chromosomes in interphase is recapitulated by simulation

**Fig. 1:**
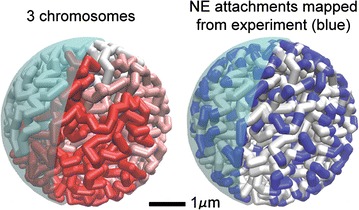
“Beads-on-string” model of *D. melanogaster* interphase chromosomes used here. On the left, the beads are colored by the three major chromosomes in wild-type *D. melanogaster*. On the right, the coloring is by Chr–NE attachments mapped from experiment, shown in blue

**Fig. 2:**
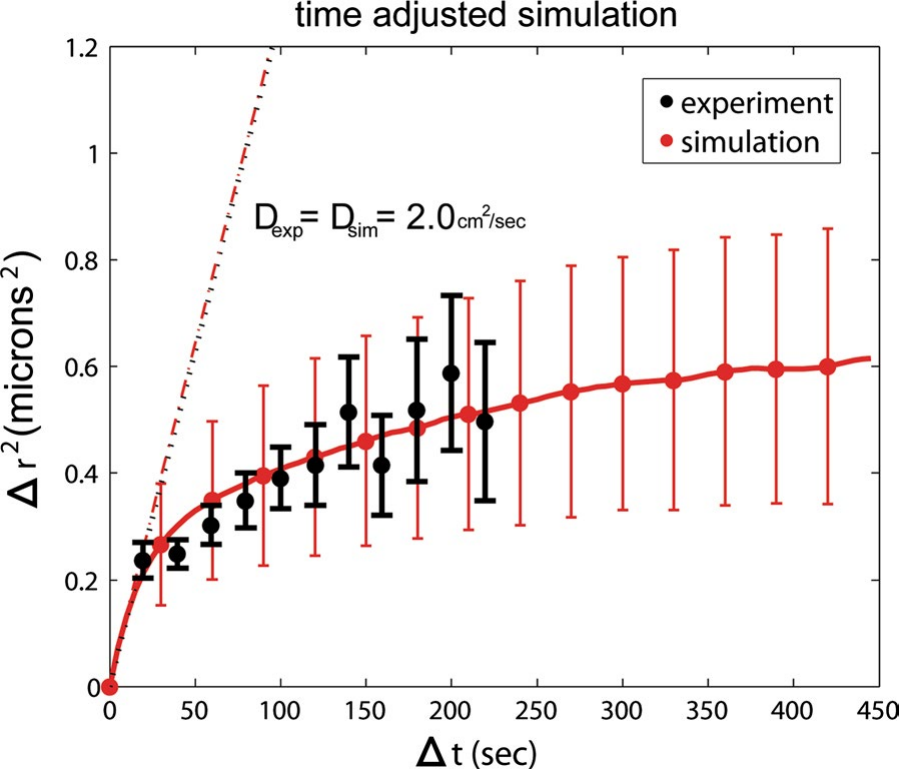
Average displacement of a chromosome locus from simulation and experiment [31]. By matching just one model parameter to experiment, simulation reproduces the complexity of experimental diffusive motion of non-polytene interphase chromosomes in the nucleus. Trivial unconfined diffusive motion would correspond to a straight line ∆*r*^2^ = 6*Dt*. Error bars on simulation show the range of motion from *n* = 8 independent trajectories. Experimental error bars are from Ref. [31]

In this work, we have developed a “beads-on-string” model of *D. melanogaster* interphase chromosomes (Fig. 1). The first test of the model is whether it can reproduce the diffusive motion of interphase chromosomes in the nucleus seen in experiment [32]. The results of this test are shown in Fig. 2: By adjusting a single parameter of the model to match the experimental diffusion coefficient, we automatically match the fairly complex, non-trivial dynamic behavior of regular interphase chromosomes under nuclear confinement. Specifically, as detailed in “Methods” section, we use a single scaling parameter, *λ*, to establish realistic timescales in each simulation (i.e., to convert the simulation time into real time). The approach is designed to match the chromosomal diffusion constant in simulation (*D*_sim_) and experiment (*D*_exp_). These diffusion constants are determined only by the initial slope of the $$\left\langle {\Delta \overset{\lower0.5em\hbox{$\smash{\scriptscriptstyle\rightharpoonup}$}} {r}^{2} \left( t \right)} \right\rangle$$ plot (see Fig. 2). Our simulations not only reproduce the initial slope, which would be trivial, but also apparently reproduces the more complex experimental diffusive motion of interphase chromosomes in the nucleus. This means that by multiplying the elapsed time in each simulation trajectory by $$\uplambda$$, we recover realistic experimental timescales; see “Methods” section.

In what follows, we test the effects of Chr–NE attachments by comparing simulations of our wild-type model (which possess Chr–NE attachments) to our control model (which lacks Chr–NE attachments).

### Chr–NE attachments may reinforce chromosome territories

**Fig. 3:**
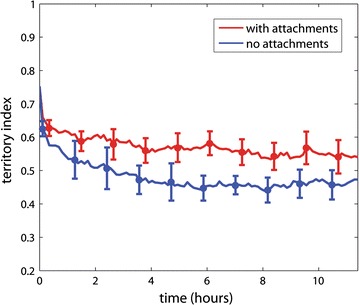
Effect of Chr–NE attachments on chromosome territories. Error bars represent 1 standard deviation calculated from *n* = 8 simulation trajectories. Red line—mean with attachments; blue line—mean without attachments. Biologically speaking, the territory index (*y*-axis) represents the fraction of chromatin inside its native convex hull (see “Methods” section)

Theoretical studies suggest that fractal-like polymer configurations are highly territorial in the sense that chromosomes occupy distinct mutually exclusive domains without entangling [11, 13, 15, 16]. On the contrary, equilibrium configurations are expected to be less organized and highly entangled [13]. Thus, transition from fractal-like to equilibrium configurations should be accompanied by the deterioration of chromosome territories. We compare chromosome territories in our wild-type model (with Chr–NE attachment) to our control model (without attachments) using an established metric called the “territory index” based on the convex hull, (see “Methods” section). Chromosomes in all simulations begin in a fractal-like configuration and are highly territorial by construction. We observe that the average territory index decreases with time in both the wild-type model and control model (Fig. 3). The simulation time was similar to the duration of *S* phase in Kc cells, which lasts ~ 10 h [33]. Thus, Chr–NE attachments are not sufficient to completely prevent deterioration of chromosome territories from their initial fractal-like configurations. However, the absence of attachments in the control model simulations leads to a faster decay in the territory index. Indeed, from Fig. 3, it is immediately clear that Chr–NE attachments slow down territory deterioration. To test whether attachments also affect the end values on biologically relevant scales, the simulation times were extended by a factor of 10 (results not shown), which takes them well beyond the experimental duration of the interphase. The end values with and without the attachments were still different. In conclusion, NE attachments may help maintain chromosome territories on biologically relevant timescales.

We add an intuitive explanation for the observation that Chr–NE attachments reinforce chromosome territories with robust dimension-based arguments. We consider two limiting cases: (1) a completely spherical chromosome territory lacking any Chr–NE attachments and (2) a chromosome territory completely anchored to the NE possessing many attachments. We propose that in (1) the convex hull representing a chromosome territory (e.g., blue chromosome in Fig. 9 below) is easily invaded by a neighboring chromosome (e.g., red chromosome in Fig. 9) due to its relatively large surface area in contact with neighboring territories. Next consider fully anchoring each chromosome to the NE, corresponding to limiting case (2). Now each chromosome territory occupies a thin layer annealed to the 2D interior of the NE. In this limiting case, the convex hull representing each chromosome territory would resemble a flattened disk that can be invaded by other chromosomes only along its 1D perimeter. In other words, there is less opportunity for a chromosome to invade neighboring chromosome territories when confined to the 2D surface of the NE. Although chromosome territories in reality adopt far more complex shapes, we argue that a chromosome partially “annealed” to the nuclear periphery by Chr–NE attachments bears some of the limiting case (2) characteristics, and thus is still less likely to be invaded by its neighbor chromosomes compared to the scenario without NE attachments.

### Chr–NE attachments limit, but do not prevent, chromosome entanglement

**Fig. 4:**
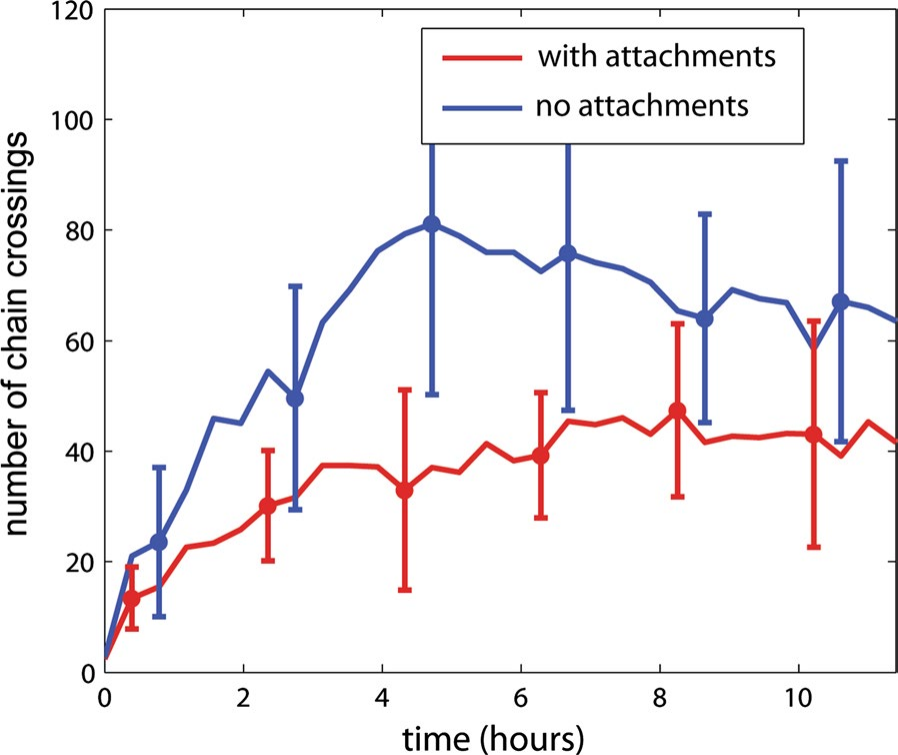
Effect of Chr–NE attachments on chromosome entanglement. Red line—mean with attachments; blue line—mean without attachments. Error bars represent 1 standard deviation calculated from *n* = 8 simulation trajectories

We investigated whether Chr–NE attachments prevent or at least delay the onset of chromosome entanglement during transition to the equilibrium state. (See “Methods” section.) Our results suggest that Chr–NE attachments delay but do not prevent the chromosome entanglement that arises during transition from FG-like initial configurations to equilibrium (Fig. 4). We note that the initial rate of accumulation of entanglement is not very different in the wild-type model (which possess attachments) and in the control model (which lacks attachments). Due to this rapid accumulation of entanglement in both models, it is possible that some chromosomes entanglement is inevitable regardless of Chr–NE attachments. We propose several conclusions. Chr–NE attachments may delay chromosome entangling long enough to ensure that chromosome separates during cell division; possibly, this delay prevents a critical amount of entanglement that would otherwise interfere with proper cell division. However, if even a minimal amount of chromosome entanglement interferes with cell division, then additional mechanisms must be enlisted to prevent entanglement more effectively than Chr–NE attachments alone. These additional mechanisms could include DNA cross-links [13]. Theoretical studies demonstrate that cross-links, which represent reversible protein-bound DNA interactions, can significantly prolong the lifetime of the fractal chromosome configurations [13]. On the contrary, limiting chromosome entanglement may not be the primary or even necessary role of Chr–NE attachments if chromosome entanglement is not a significant obstacle during the cell cycle.

### Chr–NE attachments do not help maintain the Rabl configuration

**Fig. 5:**
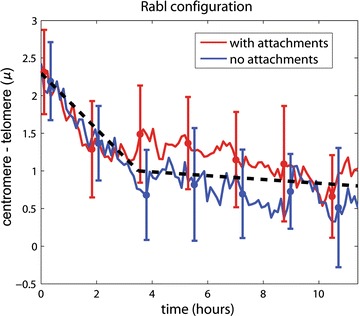
Effect of Chr–NE attachments on the degree of chromosome polarization (Rabl configuration). Error bars represent 1 standard deviation calculated from *n* = 8 simulation trajectories. Red line—mean with attachments; blue line—mean without attachments. The black dashed line is a guide to emphasize the breaking down of the Rabl configuration

All simulated chromosome were initially configured in polarized arrangements consistent with the Rabl chromosome configuration present in *D. melanogaster*. In general, the Rabl configuration is not a signature of fractal-like chromosome configurations; this is an additional property that was specifically included in the initial configurations of our model. (See “Methods” section). In the case of *D. melanogaster*, the dynamics of the Rabl configuration is well-studied experimentally; nuclei display a Rabl configuration only temporarily after mitosis [30]. Experimentally, breaking down of the Rabl configuration generally occurs ~ 2 h after mitosis with clustering of pericentric heterochromatin and euchromatic arms often occurring after 5 h [30]. For each simulation, the Rabl chromosome configuration was characterized quantitatively by exploring the axial distance (∆*r*_*z*_) between centromeres and telomeres of each chromosome arm. Our simulations suggest that breaking down of the Rabl configuration occurs after 2–4 h (Fig. 5), in relatively good agreement with experiment; the black dashed line (Fig. 5) serves as a guide to the eye that emphasizes the breaking down of the Rabl configuration occurring at 2–4 h. There was no significant difference between the wild-type model (which includes Chr–NE attachments) and control model (which lacks Chr–NE attachments). Thus, Chr–NE attachments may not prevent the breaking down of the Rabl configuration.

### Chr–NE attachments do not inhibit the deterioration of the compactness (fractal character) of the chromosomes

**Fig. 6:**
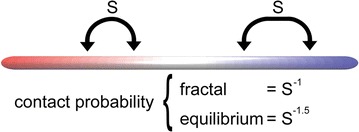
Probability of contact, *P*, between loci belonging to the same chromosome depends on their separation, *s*, along the polymer backbone. In general, this relation is captured by the parameter $\alpha$ in the expression, $P(s) = s^{\upalpha}$

**Fig. 7:**
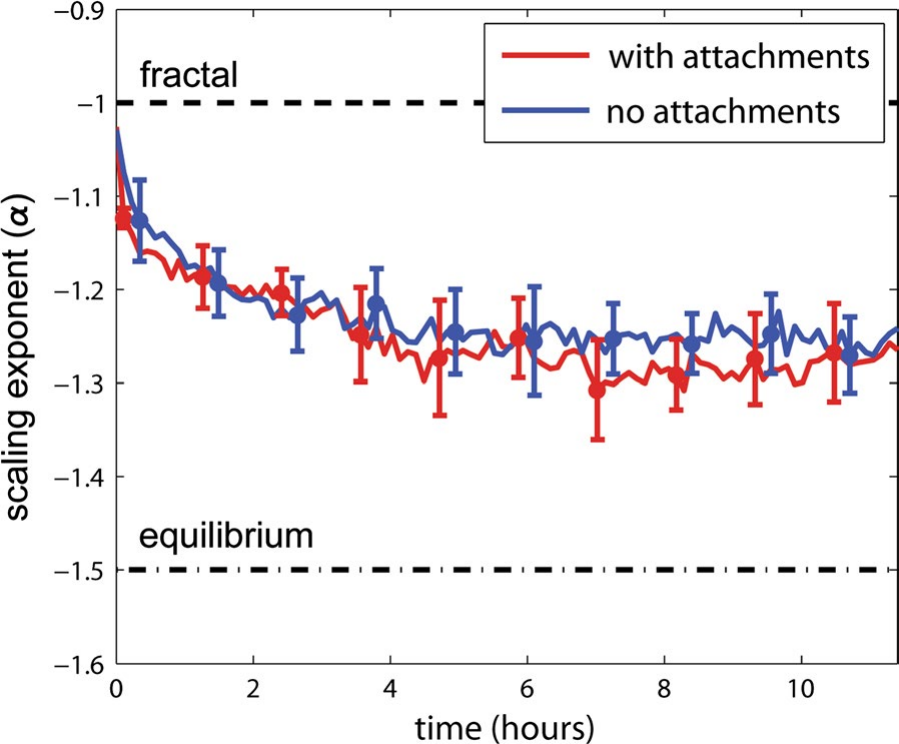
Scaling of chromosome contacts in the presence and absence of attachments to nuclear envelope. Error bars represent 1 standard deviation calculated from *n* = 8 simulation trajectories. Red line—mean with Chr–NE attachments; blue line—mean without Chr–NE attachments

As mentioned in the Introduction, the degree of chromosome compactness can be characterized by how probability of contact, *P*, between loci belonging to the same chromosome depends on their separation, *s*, along the polymer backbone (Fig. 6). In general, this relationship is captured by the parameter $\alpha$ in the expression, $P(s) = s^{\alpha }$. For fractal-like chromosome folding $\alpha = - 1$ [13]; for equilibrium chromosome folding $\alpha = - 3/2$ [13]. To determine whether Chr–NE attachments prolong the compact fractal-like chromosome folding, we plot $\alpha$ during long simulations of our wild-type model (which possesses Chr–NE attachments) versus control model (which lacks Chr–NE attachments) (Fig. 7). These long simulations represent approximately ~ 11 h of real time. (See simulation time rescaling in “Methods” section.) Since all simulations are initialized in fractal-like configurations with $\alpha \sim - 1$, we expected $\alpha$ to gradually approach the equilibrium value, $- 3/2$. Interestingly, the equilibrium value, $- 3/2$, was not reached in simulations that represent ~ 10 h in reality (Fig. 7), consistent with the very long relaxation time of fractal-like chromosome configurations reported in several previous studies [13, 17]. In our simulations, we observe that $\alpha$ decays at a similar pace regardless of the presence or absence of Chr–NE attachments (Fig. 7). Consequently, we conclude that Chr–NE interactions do not affect fractal-like chromosome scaling, which characterizes its compactness. This conclusion suggests that the effects of Chr–NE attachments on chromosomes folding are distinct from previously studied effects of chromosome cross-links [13]. Specifically, chromosome cross-links are known to greatly delay the transition to equilibrium [13]. To some extent Chr–NE attachments can be thought of as a type of cross-link—one that tethers chromosomes to a nuclear structure. Thus, it may be surprising at first that the presence of Chr–NE attachments do not affect the decay of the fractal-like scaling exponent; an explanation is offered below.

The relative insensitivity of the fractal-like scaling exponent to the presence of Chr–NE attachments can be rationalized by a simple dimensionality argument. The argument proposes that chromosome folding in the nucleus possess two limiting cases: a 2D case and a 3D case. Chromosomes with numerous NE attachments are essentially annealed to the inner NE surface and represent the 2D case; on the other hand, chromosomes without attachments can explore the interior of the nucleus and represent the 3D case. Our simulations which lack attachments correspond to the 3D case. Meanwhile, simulations which possess attachments anchor a portion of each chromosome to the NE and represent an intermediate case. However, a previous study has shown that for fractal-like curves $P(s) = S^{{\text{ - 1}}}$, essentially regardless of the dimensionality of the space [11]. In other words, the scaling exponent is the same for the 3D case and the 2D case [11]. Thus, the “turning on” of Chr–NE attachments is expected to have little effect on the scaling exponent in simulation. Note that the invariance of the scaling exponent does not imply that Chr–NE attachments do not affect the actual probability of gene–gene contacts [29] (as a trivial example, consider multiplying *P(s)* by a constant factor).

### Robustness of the results

1. Effects of turning on Chr–NE attachments are robust to topoisomerase II activity.

We have reassessed the effects of Chr–NE attachments by comparing simulations of the wild-type and control models, *both of which were altered to exhibit the same lowered barrier to strand crossing, as detailed in* “Methods” section. The lowering of the strand crossing barrier is a simple scheme to mimic the effects of topoisomerase II: an enzyme that permits crossing of dsDNA [13]. Our main finding is that all previously stated results (above) were recapitulated with the reduced strand crossing barrier. That is we reiterate our conclusion that the presence of Chr–NE attachments has two key effects, regardless of simulated Topo II activity: chromosome territories are reinforced and chromosome entanglement is reduced. Chr–NE attachments have little effect on the chromosome scaling exponent (compaction) and the Rabl chromosome configuration, also with the simulated Topo II activity present.

(2) Effects of turning on Chr–NE attachments are robust to the presence of heterochromatin.

Our main models are based on release 5 of the D. melanogaster genome which includes its sequenced parts; however, approximately 50 Mb of heterochromatin remain unsequenced. We checked the robustness of our results by considering an additional set of more detailed “companion” models: wild-type models and control (Null) models that include ~ 50 Mb of unsequenced heterochromatin. (See “Methods” section for details.) Simulations based on these companion models reiterate the aforementioned conclusions: (a) Chr–NE attachments reinforce chromosome territories; (b) Chr–NE attachments mitigate chromosome entanglement; (c) Chr–NE attachments have little effect on the chromosome scaling exponent; and (d) Chr–NE attachments do not affect the relaxation time of the Rabl chromosome configuration. See Additional file 1: text S1 and Figures S1–S4.

(3) Effects of turning on Chr–NE attachments are robust to the strength of Chr–NE attachments.

An association between NE contact frequencies for polytene salivary gland nuclei and smoothed Lam binding values for non-polytene nuclei suggests that LADs may vary in their affinity for the NE. Despite this correspondence, the relationship between Lam binding values and affinity for the NE may be complex. Thus, the main models we consider make the simplifying assumption that all beads corresponding to LADs have the same affinity for the NE. We checked the robustness of our results to this assumption by considering more complex wild-type models and control models that vary the strength of NE interaction for each of the beads that represent LADs; see details in “Methods” section. Simulations based on this version of our companion model reiterate our four major conclusions: (a) Chr–NE attachments reinforce chromosome territories; (b) Chr–NE attachments mitigate chromosome entanglement; (c) Chr–NE attachments have little effect on the chromosome scaling exponent; and (d) Chr–NE attachments do not affect the relaxation time of the Rabl chromosome configuration. See Additional file 1: text S2 and Figures S5–S8.

(4) Effects of turning on Chr–NE attachments are also robust to initial conditions used by the simulations; see “Methods” section.

## Discussion

### Overall conclusions

Overall, our simulations of fruit fly interphase chromatin suggest an important role of Chr–NE interactions in preserving nuclear architecture in higher eukaryotes on biologically relevant timescales. We emphasize four key results: (a) Chr–NE interactions assist in prolonging chromosome territories; (b) Chr–NE interactions limit chromosome entanglement; (c) Chr–NE interactions do not inhibit deterioration of compactness (fractal character) of chromosomes; and (d) Chr–NE interactions do not prevent the breaking down of the Rabl configuration which occurs after 2 h. Each result was found to be robust to the presence of simulated topoisomerase II activity and various model details. In addition, the effects of Chr–NE interactions on chromosome territories are explained by a simple volume accessibility argument.

### Chr–NE attachments limit chromosome entanglement

We have developed a new simple measure of chromosome entanglement reminiscent of chromosome separation during the cell cycle. The metric enumerates chromosome strand crossings upon putative translation in free space. Our results suggest that Chr–NE attachments do not completely prevent chromosome entanglement in simulation. However, the accumulation of chromosome entanglement is clearly delayed in the presence of Chr–NE attachment. Importantly, our simulations suggest that the timescale of this delay is on the order of the lifetime of the cell interphase. Thus, Chr–NE attachments not only limit chromosome entanglement, they do so on biologically relevant timescales. This result is consistent with previous studies of chromosome knot complexity [13] and offers a potentially biological interpretation. In particular, the enumeration of strand crossings in the absence of Chr–NE attachment may exceed the capacity of Topo II necessary to fully separate two chromosomes. Unfortunately, little is known about Topo II activity in the interphase nucleus. An analysis of chromosome strand crossings and Topo II in the interphase nucleus will be pursued in a future study.

### Unique effect of Chr–NE attachments on different fractal-like signatures

Support for the fractal-like configurations in human and *Drosophila* stems from the experimentally observed chromosome interaction probability described by the scaling law *P*(*s*) = *s*^−1^. Physically speaking, a scaling law of this form means that chromosome loops form on all lengths scales, which rules out many equilibrium-based chromosome folding models [13]. However, this unique scaling is lost upon transition to equilibrium. If this change arises exclusively due to reptation of the polymer ends, then the transition time to equilibrium may be on the order of $\sim N^{3}$ (number of monomers), leading some computational studies to suggest that fractal configurations are stable for ~ 500 years [17]. On the contrary, other studies suggest that fractal configurations quickly transition to a semi-entangled state and that cross-links within the fractal globule are necessary to maintain its native shape [13]. The situation is even less clear in *D. melanogaster* in which fractal configurations are expected to reach equilibrium much faster due to its smaller genome size (and thus smaller $\sim N^{3}$). Although the endpoint of our simulations has unlikely reached true equilibrium, we clearly see changes in chromosome contacts (scaling), chromosome territories, and chromosome entanglement on biologically relevant timescales. In addition, our model clearly suggests that Chr–NE interactions have unique effects on the signatures typically co-associated with fractal chromosome configurations. In particular, turning on Chr–NE attachments in our simulations appears to stabilize chromosome territories with little effect on the chromosome scaling exponent.

### Chr–NE interactions do not affect the Rabl configuration

The field of research interested in 3D genome organization can perhaps be traced back to the original studies of Rabl and Boveri, which described a polarized configuration of chromosomes (now known as the Rabl configuration) and suggested the possibility of a highly organized nucleus. The configuration is present in multiple lineages of metazoans such as fruit fly [8], yeast [34], and wheat [35]; yet the details of the Rabl configuration are largely unknown. The characteristic polarization, with centromeres and telomeres at apogee within the nucleus is speculated to be a vestige of the previous anaphase, but has not been confirmed. In the case of *D. melanogaster*, the dynamics of the Rabl configuration is well-studied; its known lifetime is on the order of 2 h in interphase with apposition of telomeres and pericentric heterochromatin often occurring after 5 h [30]. Our simulations suggest that the lifetime of the Rabl configuration does not depend on the presence or absence of Chr–NE interactions. In addition, chromosome motion in our simulations is guided only by the dynamics of Brownian motion. Thus, the underlying Brownian motion of chromosomes may be sufficient to dictate large-scale chromosome motions that evolve over the course of several hours.

### Chr–NE attachments prolong chromosome territories regardless of simulated Topo II activity

The question of stabilizing territorial chromosome configurations was raised in a recent computational study that noted a rapid transition to the equilibrium state in the presence of active topoisomerase II (Topo II), an enzyme that facilitates strand crossing of the DNA [13]. Although knowledge of Topo II activity in interphase is limited, as is the ability of Topo II to pass whole strands of chromatin, current evidence suggests that Topo II does act efficiently on the nucleosome-bound DNA. Therefore, additional factors may be necessary to topologically constrain territorial chromosome configurations and prolong their lifetime in the cell. One proposal is the cross-linking of distant chromosomal loci, which prolonged the fractal globule in recent simulations [13]; our complemental proposal involves the interactions between chromosomes and the NE. These interactions have already been shown to constrain chromatin motion in vivo [32] and reinforce chromosome territories in silico [28]; both of these results allude to a topologically preserving role of Chr–NE interactions. The results presented here are consistent with these previous studies and uncover an important detail: Chr–NE interactions may stabilize chromosome territories and do so regardless of simulated Topo II activity. This detail may be critically important if future experiments confirm the activity of Topo II in the interphase nucleus at a level that would otherwise induce rapid transition of biologically relevant states (compact, fractal-like) of chromatin to equilibrium.

### The conclusions are robust to model details

The biology of real chromosomes in the cell nucleus is undoubtedly highly complex, with a myriad of details affecting biological outcomes. However, the overall behavior of chromosomes in 3D in the cell nucleus is still expected to be consistent with a relatively simple physical system: that of polymers under confinement. Polymers under confinement essentially consist of bonded monomers interacting among themselves (including implicit interaction with the solvent) and with the boundary of the system. Thus, the *basic physics* of such a system is determined chiefly by the bonded interactions between monomers, non-bonded interactions between monomers, and boundary conditions. Although real biological systems have numerous additional details—many of which are unknown—as long as one asks *general enough* questions about chromosome geometry under confinement, answers to these questions are unlikely to be affected by these details. These are the types of questions we have asked in this work. The general expectation that one can obtain reliable answers to these types of questions from a relatively simple model presented here is confirmed by the numerous robustness tests to details we have performed.

### Limitations and future work

Our model is specific to *D. melanogaster*, including the specific chromosome-to-nucleus volume ratio. Therefore, we cannot conclude that Chr–NE attachments also prolong territories in coil-like configurations which may exist in, e.g., yeast. More work is needed to determine the role of Chr–NE interactions in organisms and cell types where chromosome folding principles differ significantly from those of fruit fly. We acknowledge several more specific limitations of our computational models. In particular, the bead radius, a key parameter in our computational models, is determined by the Kuhn length of *D. melanogaster* chromosomes, which in turn limits the highest resolution of our computational models. For simplicity, here we have used short-range potentials to represent Chr–NE attachments but concede that potentials of a different form may be as realistic. For example, dynamically forming bonds between chromosomes and the NE could be used to represent the protein dependent mechanisms that physically anchor chromosomes to the NE. Still, we believe that even with these limitations, our efforts to incorporate the Chr–NE attachments in models of 3D genome organization in higher eukaryotes is likely an improvement over their absence.

## Methods

### Modeling approach

The five largest chromosome arms of *D. melanogaster* are modeled as beads-on-string [13, 36–40]. The sixth arm, chromosome 4, is not considered due to its negligible length. Each beads-on-string chromosome consists of particles interacting as soft spheres bonded by harmonic spring potentials; a detailed description of potentials is provided below. We additionally consider the presence of a nucleolus in simulations by excluding the volume of a spherical region .2 µ in radius positioned near the X chromosome centromere. Experimental data [12, 25, 32] for the chromosomes and the nucleus become realistic model parameters and constraints imposed during simulations (see Table 1 for parameters). Fractal-like initial configurations of chromosomes are assembled on a simple cubic lattice and transferred to free space during warm-up integration. Complete details of model assembly and warm-up protocol are provided below. Simulations are performed in Espresso [41]. The “beads-on-string” model of *D. melanogaster* interphase chromosomes is depicted in Fig. 1.

**Table 1:** Essential model parameters

Parameter	Value
X chromosome	22,422,827 bp = 321 beads
2L arm	23,011,544 bp = 329 beads
2R arm	21,146,708 bp = 302 beads
3L arm	24,543,557 bp = 351 beads
3R arm	27,905,053 bp = 399 beads
Nucleus radius ($R_{\text{nuc}}$)	2.25 µm
Bead radius ($R_{\text{bead}}$)	.1 µm
Bead mass	77 M daltons
% confinement	$(1702 \cdot R_{{_{\text{bead}} }}^{3} )/R_{\text{nuc}}^{3}$ .15

### Bead size and chromosome persistence length

It is well known from polymer physics that a chain with persistence length, $l_{p}$, may be modeled as a self-avoiding walk (SAW) segmented by the Kuhn length, $l_{k} = 2l_{p}$. Although the Kuhn length of interphase chromatin has not been directly measured, several previous studies [11] estimate the Kuhn length based on the following argument which we briefly reiterate. The persistence length of double-stranded DNA is known to be 150 bp, and the linker DNA between histones is on average 50 bp [42–45]. Since the histone bound DNA consists of 150 bp and does not contribute to the flexibility of the chromatin, the Kuhn length of the chromatin corresponds to about six histone/linker segments amounting to 1200 bp. The estimated persistence length of 600 bp is a lower bound for the following reason: Protein bounds to DNA and possible higher-order structure of chromatin will increase persistence length. Indeed, persistence length estimates of the yeast 30-nm fiber range as high as 40,000 bp [46]. The aggregate of multiple other experiments [47, 48] suggest the persistence length of chromatin ranges from 3000 to 20,000 bp. The details of chromatin packaging specific to *D. melanogaster* are limited and complicated by evidence of chromatin remodeling which can affect the chromatin flexibility [49]; therefore, we conservatively take each bead in our model to represent 70,000 bp (which is more than twice the persistence length measurements of most experiments) and model the chromatin as freely jointed beads-on-string. Next, we calculate the mass and volume of each bead, which is important for establishing the simulation time step (discussed below). The 70,000 bp represented by each bead is associated with approximately 350 nucleosomes. Since the mass of each nucleosome is ~ 100,000 Da and each base pair ~ 600 Da, we assign each bead in our model a mass, *m*_bead_, of 77 MDa. To establish the diameter of each bead, *D*_bead_, we first approximate each nucleosome as a cylinder with radius and length of 5 nm [11]; its volume, *V*_n_, is therefore π∙5^3^ cubic nanometers. The diameter of each bead depends on the volume and arrangement of the 350 nucleosomes it represents. The 350 nucleosomes represented by each bead are likely structured as a 10–30-nm fiber which sets the diameter of each bead at ~ .2 microns.

### Volume of nucleus and chromatin

We take the nucleus to be approximately spherical with a diameter of 4.5 µm; thus the 1702 beads in our model occupy ~ 15% of the nuclear volume as in several previous studies [17, 19, 50]. A summary of essential model parameters is shown in Table 1.

### Details of the potential function and simulations

Five equations are used to model dynamics of the bead-spring system (see below). These include a harmonic potential between bonded beads, a pure repulsive Lennard-Jones potential for non-bonded beads, a Lennard-Jones cosine-modulated potential for beads that map to lamin-associated domains, a pure repulsive Lennard-Jones potential for beads not mapping to lamin-associated domains, and the Langevin equation of motion. The novelty of this approach is the use of a Lennard-Jones cosine potential to anchor specific beads to the periphery (spherical boundary) of the system that represents the NE. The harmonic potentials (for bonded interactions) and pure repulsive Lennard-Jones potentials (for non-bonded interactions) are widely used in polymer physics [13, 36–40]. This simple approach incorporates the fundamental properties of confined polymer systems: bonded interactions, non-bonded interactions, and boundary conditions. Although real biological systems have numerous additional details—many of which are unknown—the same fundamentals govern dynamics of chromosomes confined within the nucleus (see also our discussion). Parameters of the equations described below are provided in Table 2. Temperature of 300 K is used throughout the study.

**Table 2:** Simulation parameters

Equations	Parameter	Value
1	$\kappa$	$10K_{\text{b}} T$ (reduced to $1K_{\text{b}} T$ for Topo II simulations)
1, 2, 3, 4	$\ell$	.2 microns (twice the bead radius)
2, 3, 4	$\sigma$	$\frac{\ell }{{2^{{{1 \mathord{\left/ {\vphantom {1 6}} \right. \kern-0pt} 6}}} }}$ where $\ell = .2$ as in Eq. 1^a^
2	$\varepsilon$	$3K_{\text{b}} T$
3	$\alpha$	$\pi \left[ {\left( {6^{{{1 \mathord{\left/ {\vphantom {1 6}} \right. \kern-0pt} 6}}} \sigma } \right)^{2} - \ell^{2} } \right]^{ - 1}$
3	$\beta$	$\pi - \ell^{2} \alpha$
3, 4	$\xi$	$3K_{\text{b}} T$
5	*m*	77MDa (bead mass)
5	*γ*	$\tau^{ - 1}$ where $\tau = \sigma \sqrt {{m \mathord{\left/ {\vphantom {m \varepsilon }} \right. \kern-0pt} \varepsilon }}$

^a^To model the excluded volume of the nucleolus we use $\ell = .3$ microns which is a sum of the bead radius and nucleolus radius

#### Bonded interactions

All bonded bead–bead interactions are modeled with a harmonic potential (Eq. 1). Here $r_{ij}$ specifies the distance between bead *i* and bead *j*. An energy barrier of 10*k*_b_*T* prevents chain crossing by prohibiting significant bond fluctuations that would otherwise permit one link in the bead-spring chain from extending sufficiently to cross over another.

1 $$u\left( {r_{ij} } \right) = \frac{1}{2}\kappa \left( {r_{ij} - \ell } \right)^{2}$$

#### Non-bonded interactions

All non-bonded bead–bead interactions (as well as the excluded volume of the nucleolus) are modeled with a pure repulsive Lennard-Jones potential (Eq. 2). Here $r_{ij}$ specifies the distance between bead *i* and bead *j*.

2 $$u\left( {r_{ij} } \right) =\left\{\begin{array}{ll}4\varepsilon \left[ \left( {\frac{\sigma }{{r_{ij} }}} \right)^{12}- \left( \frac{\sigma }{{r_{ij} }} \right)^{6} + \frac{1}{4}\right], & r_{ij} \le \ell \\0, & r_{ij} > \ell \end{array}\right..$$

#### Boundary condition 1

**Fig. 8:**
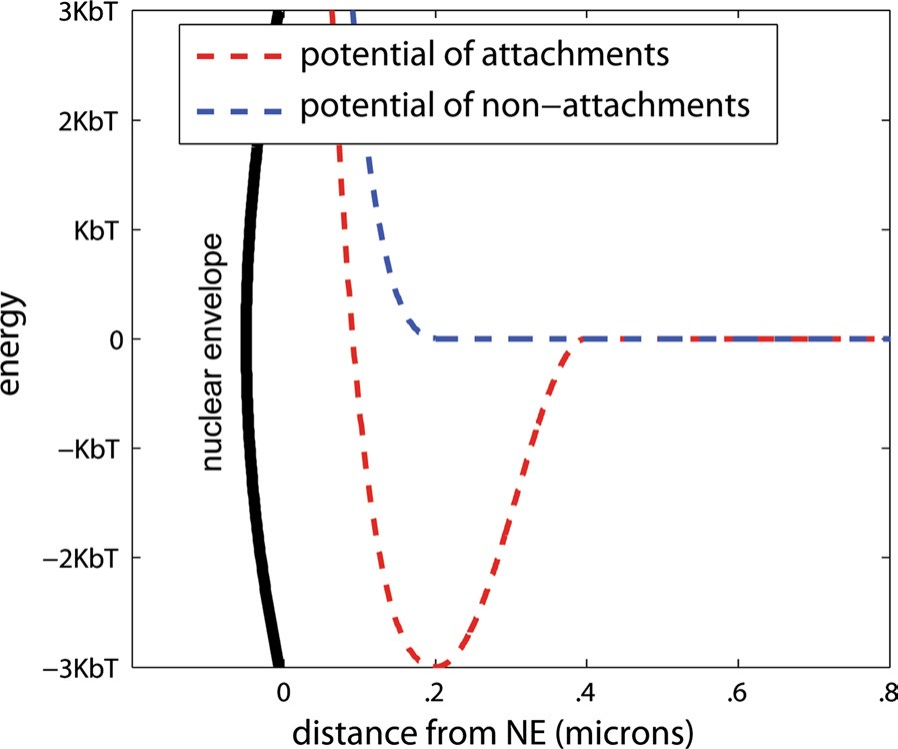
Model of Chr–NE interactions. Specific beads are attached to the NE using a Lennard-Jones cosine interaction, Eq. 3. Beads lacking affinity for the NE use a shifted Lennard-Jones potential, Eq. 4

Experimental Lam data [25, 26] for *D. melanogaster* interphase chromosomes are mapped to the corresponding beads in our model. These beads interact with the NE in simulation via short-range, attractive Lennard-Jones “cosine” interaction (Eq. 3). Here $r_{i}$ specifies the distance between bead *i* and the nuclear envelope. In this scheme, the minimum of a traditional Lennard-Jones interaction is smoothly stitched to zero to maintain the potential function’s differentiability [41]. In the main model, the $\xi$ parameter is set to 3*k*_b_*T* (see Table 2) meaning that NE affinity is e^3^ ~ 20 times higher for attachment beads than non-attachment beads. This choice of the affinity difference is made to match the strength of chromosome–NE attachments in the fruit fly nucleus. Specifically, previous studies of polyene chromosomes in fruit fly demonstrated that probability of NE attachment in an ensemble of nuclei ranges between approximately 0.03–0.6 [8]. Thus, a 20-fold (0.6/0.03) difference was observed for chromosome contact frequencies in the polyene nucleus, and this range was reiterated for non-polytene interphase chromosome [25]. As a corollary, beads representing Lam-associating regions “anchor” to the NE by being partially confined to the potential well (red in Fig. 8).

3 $$\begin{aligned} u(r_{i} ) = \left\{ {\begin{array}{*{20}l} {4\xi \left[ {\left( {\frac{\sigma }{{r_{i} }}} \right)^{12} - \left( {\frac{\sigma }{{r_{i} }}} \right)^{6} } \right]} \hfill & {0 < r_{i} \le \ell } \hfill \\ {\frac{1}{2}\xi \left( {\cos \left[ {\alpha r_{i}^{2} + \beta } \right]} \right) - 1} \hfill & {\ell < r_{i} \le 6^{{{1 \mathord{\left/ {\vphantom {1 6}} \right. \kern-0pt} 6}}} \sigma } \hfill \\ 0 \hfill & {r_{i} > 6^{{{1 \mathord{\left/ {\vphantom {1 6}} \right. \kern-0pt} 6}}} \sigma } \hfill \\ \end{array} } \right.. \hfill \\ \hfill \\ \end{aligned}$$

#### Boundary condition 2

For beads that do not map to LADs, interactions with the NE are modeled with a pure repulsive Lennard-Jones potential (Eq. 4). Here $r_{i}$ specifies the distance between bead *i* and the NE. See also Fig. 8 (blue dashed line).

4 $$\begin{aligned} u\left( {r_{i} } \right) = \left\{ {\begin{array}{*{20}l} {4\xi \left[ {\left( {\frac{\sigma }{{r_{i} }}} \right)^{12} - \left( {\frac{\sigma }{{r_{i} }}} \right)^{6} + \frac{1}{4}} \right],} \hfill &\quad {r_{i} \le \ell } \hfill \\ {0,} \hfill &\quad {r_{i} > \ell } \hfill \\ \end{array} } \right.. \hfill \\ \hfill \\ \end{aligned}$$

#### Dynamics

The simulation of a bead-spring polymer model is typically implemented with the Langevin equation [51–54]. Here **U** is the sum of potential energy terms (Eqs. 1–4), and *x* represents the positions of beads in the model.

5 $$m\ddot{x} = - \nabla U -\upgamma\dot{x} +\Gamma \text{.}$$

In this scheme, a viscous friction, controlled by the value of *γ*, is balanced by uncorrelated Gaussian noise, Γ, which represents collisions with the environment, e.g., solvent molecules. The Langevin approach is well justified empirically and theoretically. Within the approach, the dynamics of each bead is governed by the bead–bead interactions and interaction with the solvent, including the effect of solvent viscosity. Since the polymer is coarse-grained, it is safe to assume that the timescales corresponding to the oscillation of the bead in the potential wells of either the connecting springs or the non-bonded interactions are much longer than the time between consecutive collisions of solvent molecules. This is what allows solvent collisions to be modeled as random uncorrelated noise, Γ. Indeed, empirical observations of GFP tagged loci have confirmed that chromosome dynamics in interphase is Brownian [32].

#### Time step

We use the integration time step  $t_{\text{step}} =\uptau/100$; here  ${\tau = \sigma }\sqrt {m /\upvarepsilon}$ is the Lennard-Jones (LJ) timescale [55]. Temperature is maintained by setting the friction term, (*γ* in Eq. 5) to $\tau^{ - 1}$ as in Refs. [56, 57]. This choice of *γ* is discussed in detail below.

#### Simulation parameters

Parameter values in Tables 1 and 2 were used for all simulations described in the main text as well as the companion models (see supplementary material). Parameter changes for companion models and those that consider the effects of topoisomerase II (Topo II) are described below.

### Simulation timescales

In Langevin’s original 1908 paper [54], the viscous resistance constant, *γ*, was determined by the Stokes’ formula ($\gamma$ = *6πµa*) instead of the Lennard-Jones (simulation) time. In the Stokes formula, *µ* is solvent viscosity and *a* is the radius of the Brownian particle. Often in coarse-grained polymer models, this approach implies a large value of *γ* and thus large viscous resistance forces compared to the bonded and non-bonded interactions in simulation. In our case, this choice would severely limit the simulation time step because the large viscous resistance forces would require a small time step on the order of $1/\gamma$. The modern approach [55, 56, 58, 59] is to allow the Lennard-Jones forces to dictate the effective simulation times scales by artificially setting $\gamma$ to the inverse of the LJ timescale *τ*, (see above). This artificial lowering of *γ* grants a larger time step without affecting the thermodynamic sampling of polymer configuration space. The use of a low Langevin collision frequency is a technique [60–62] often used in modern atomistic MD simulations to speed up conformational sampling, e.g., of protein folding [60–62]. Simply setting *γ* to the inverse of the Lennard-Jones (simulation) time is common in polymer simulations [55, 56, 58, 59].

Essentially, artificially reducing $\gamma$ implies abandoning realistic timescales in a simulation in favor of rapidly exploring the available configuration space [60–62]. In fact, a simulation that combines coarse graining and $\gamma$ reduction can exceed the finite lifetime of most cells in interphase [17]. However, without knowing how timescales of such a simulation map to reality, it is impossible to predict what happens on biologically relevant timescales. Fortunately, we can use experimental data [17, 40] for the mapping, that is to establish, a posteriori, a correspondence between the simulation and experimental timescales. The approach is based on the experimental observation that for wild-type *D. melanogaster*, chromosome motion is diffusive: a fluorescently tagged chromosomal locus experiences Brownian motion under confinement [32]. For free Brownian particles, the mean-squared displacement in 3D follows the Einstein equation $\overrightarrow {\Delta r}^{2} = 6{Dt}$, while in the presence of confinement by NE $\left\langle {\Delta \vec{r}^{2} (t)} \right\rangle$ eventually plateaus. Both these features, Fig. 2, show a biological interpretation: The initial slope of $\left\langle {\Delta \vec{r}^{2} (t)} \right\rangle$ plot reflects the rate of unimpeded diffusion where individual displacements of the locus are small and the confinement effects are minimum; the plateau height reflects the radius of the volume accessible to the tagged loci within the nucleus. Thus, we can estimate the diffusion constant in simulation, *D*_sim_, or experiment, *D*_exp_, from the initial slope of the corresponding $\left\langle {\Delta \vec{r}^{2} (t)} \right\rangle$ plot. (The radius of confinement in simulation is approximated by the ordinate at which the $\left\langle {\Delta \vec{r}^{2} (t)} \right\rangle$ plot levels off.) In the case of wild-type *D. melanogaster*, experimentally estimated [32] diffusion coefficient $D_{\exp } = 2 \cdot 10^{ - 11} \frac{{{\text{cm}}^{2} }}{\text{s}}$. This parameter is used to map simulation trajectories to experimental timescales as described below.

*To establish the correspondence between the simulation and experimental timescales,* we begin with a plot of $\left\langle {\Delta \vec{r}^{2} (t)} \right\rangle$ for our wild-type model and determine its initial slope, *6D*_sim_ (see Fig. 2). Next, we define a dimensionless parameter, λ, such that *λD*_sim_ = *D*_exp_, where *D*_exp_ is taken from experiment. Thus, if we rescale the simulation time with the fitting parameter *λ*, the experimental rate of diffusion is reproduced. Our results demonstrate that the simulation not only reproduces the initial slope, which would be trivial, but also reproduces the more complex experimental diffusive motion of interphase chromosomes in the nucleus, Fig. 2. Thus, we are reasonably confident that the simulation time rescaled by *λ* corresponds to the realistic experimental time. In the simulations reported here, the value of *λ* was not identical between individual trajectories, but typically fell in a narrow range between 0.4 and 0.45.

### Models we consider

#### Main models

These models are based on release 5 of the* D. melanogaster* genome which includes its sequenced parts: all euchromatin and some heterochromatin. We consider two versions. A *wild-type model* possesses all known parameters of *D. melanogaster* nucleus and includes the Chr–NE attachments identified experimentally in [25]; this model corresponds to the experimentally accessibly wild-type. The second one is the *control model* in which chromosomes do not possess specific sites of attachments to the NE. This model possess all other features of the wild-type model and represents a hypothetical mutant in which chromosomes do not anchor to the NE. Equations and parameters to reproduce these models are provided in Tables 1, 2 and Eqs. 1–5.

### Justification of several specific model details

In our modeling, we have used the DamID data from cultured cells. The specific Kc cell line was created from disaggregated 8–12-h-old embryos [63], and cells are up to 90% diploid and female—these features match those of our model [64, 65]. The chosen cell size seen in our diffusion modeling, Fig. 2 (the limiting value of the locus displacement, which is limited by the nucleus size), agrees with the experiment [32]. Perhaps most importantly, the chromatin-to-nucleus ratio in our simulations is about 0.15, again in agreement with multiple previous studies [17, 19, 50]. It is this ratio that plays a key role in the general polymer properties of chromatin [13].

A recent Hi-C study has demonstrated A/B compartmentalization, where domains in compartment A interact mostly with other type A domains, and vice versa [11]. Unlike mammals, which have radial positioning of chromosomes within the nucleus [66].* Drosophila* chromosomes have initial Rabl orientation that persists ~ 2 h after mitosis in the nucleus [30]. Due to Rabl orientation of the chromosomes in fruit fly, any part of any chromosome can potentially attach to the periphery. As a result, active (A) and inactive (B) TADs are frequently intermingled along the chromosomes and nuclear space [11]. All Chr–NE interactions (or LADs) in our model are taken from experimental data on fruit flies; these interactions would roughly be corresponding to “B” compartment [25, 26]. Beads that do not interact with the lamina would roughly represent “A” in our model.

In contrast to mammals, non-polytene chromosomes in fruit fly pair in interphase as do polytene chromosomes because strong somatic synapsis exists [67]. For this reason, we modeled the fruit fly chromosomes as haploid. Similarly, we previously modeled polytene chromosomes as a haploid set consisting of five major arms just as they look under a microscope. In the present model, we assume that each bead contains fragments of both homologous chromosomes.

#### Companion models with 50 Mb of unsequenced heterochromatin

These models are based on release 5 of the *D. melanogaster* genome plus another 50 Mb of heterochromatin that remains unsequenced [68]. The heterochromatin was modeled with an additional region of excluded volume set to 3 µ^3^. Here, the amount of excluded volume (3 µ^3^) was designed to match the combined volume of 714 beads which in turn represent 50 Mb of chromatin in our models. The heterochromatin was placed at the nuclear periphery in close proximity to the clustered chromosome centromeres with its shape that of a positive meniscus lens. The nucleus radius for the companion models was set to 2.37 µ to maintain the same chromosome volume to nucleus volume ratio used in the main models. All other parameters in Table 1 remained the same; all parameters in Table 2 remained the same. Equations 1–4 did not change. Again, we considered two versions: a *wild-type model* with Chr–NE attachments taken from a recent study that identified 412 *Drosophila* LADs [26], and a *control (Null) model* in which chromosomes do not possess specific sites of attachments to the NE.

#### Companion model with varied strength of Chr–NE attachment

In this model, the strength of NE interactions is varied for each of the beads that represent LADs. Specifically, the $\xi$ parameter in Eqs. 3–4 (corresponding to the well depth for the Lennard-Jones “cosine” interaction) is set to one of three random values for each bead: 2*K*_b_*T*, 3*K*_b_*T*, or 4*K*_b_*T*. These values approximately represent the range of contact frequencies in polytene salivary gland nuclei. Briefly, a well depth of 2*K*_b_*T* means that NE affinity is e^2^ ~ 7 times higher for attachment beads than non-attachment beads. Well depths of 3*K*_b_*T* and 4*K*_b_*T* imply NE affinity 20 and 50 times higher than non-attachment beads, respectively. We compared simulations based on *wild-type models* and *control (Null) models as before*. In the wild-type models, the chromosome–NE attachments are taken from a recent study that identified 412 *Drosophila* LADs [26]; the control models do not possess specific sites of attachments to the NE. All other parameters are from Tables 1 and 2 and are the same as used for the main model.

#### Models with simulated topoisomerase II activity

Topoisomerase II (Topo II), an enzyme that facilitates strand crossing of the DNA, may increase the relaxation time of fractal-like chromatin configurations by allowing strands of dsDNA to cross. Topo II activity in interphase is unclear: Some studies indicate that Topo II is present in the interphase nucleus [32], while others suggest that most of the Topo II is degraded upon exit of mitosis [40]. Regardless, we test the robustness of our results to topoisomerase II by performing simulations in which the default strand crossing barrier ($\upkappa$ in Table 2) is reduced by a factor of ten. The idea of this simple approach to modeling topoisomerase II activity, fully implemented here, was proposed previously in Ref [13]. Again, we have considered two versions of the model with the topo II activity present: A *wild-type model* with chromosome–NE attachments and a *control (Null) model* without chromosome–NE attachments. The outcome of these simulations is detailed in Results.

### Definition of fractal-like configurations

All simulations are initialized in “fractal-like” configurations. These fractal-like configurations are designed to match two key features possessed by experimental *D. melanogaster* chromosomes. First, the decay of intra-chromosomal contacts is described by the power law, $P(s) = s^{ - 1}$, where $P$ is the probability of contact between two beads belonging to the same chromosome, and *s* is their separation along the polymer backbone. Second, chromosomes are territorial and lack entanglement. In addition to these two key fractal-like signatures, chromosomes in fruit fly [6–8] possess a distinctly polarized (Rabl) chromosome arrangement characterized by clustering of chromosome centromeres and telomeres at opposite ends of the nucleus. These characteristic arrangements are not encoded automatically in the initial “fractal-like” configurations. Nonetheless, the Rabl (polarized) chromosome configuration is enforced in our model as an additional constraint by the initial positioning of chromosome centromeres and telomeres at opposite nuclear poles. The effects of Chr–NE attachments are then studied by comparing the dynamics of the wild-type and control models as each transition to the equilibrium state. This aim is made quantitative by computing four observables during simulation trajectories: the chromosome scaling exponent, the chromosome territory index, the rates of chromosomal diffusion, and chromosome entanglement. Persistence of the Rabl chromosome configuration is compared to the 2-h relaxation time suggested by experiment [30]. Simulations are mapped to biologically relevant timescales (see “Results” section) and checked for robustness to the specifics of chain crossing and model details.

### Generation of the initial fractal-like configurations

Two well-known classes of space-filling curves embody the key properties of “fractal-like” configurations: Peano curves and Hilbert curves [69]. To generate the initial fractal-like configurations used in simulation, we begin with a precomputed Peano space-filling curve on a three-dimensional 128 × 128 × 128 unit lattice. We used MATLAB (version R2016a) to generate this precomputed curve. Three steps are then used to generate the three chromosomes. Step 1: we select a contiguous segment of the precomputed Peano curve at random for each arm. Step 2: we apply a Monte Carlo procedure to each arm that enforces the Rabl chromosome configuration. Step 3: the right and left arms of chromosomes two and three are attached and folded. The initial configuration is completed with an additional step (Step 4) that repositions the three chromosomes on the ambient lattice and rescales each to match the size of beads in simulation. Each step is described in additional detail below. An example of the initial fractal-like configuration is shown in Additional file 1 Figure S9 panel A.

Step 1 details: The number of lattice units for each of contiguous random segment of the Peano curve matches the number of beads for each arm, respectively.

Step 2 and 3 details: The Monte Carlo procedure designed to enforce the Rabl chromosome configuration of each arm has three phases. In the first phase, putative rotations are applied that elongate the arm parallel to the *Z*-axis of the lattice. A single putative rotation, $\theta_{i}$, consists of selecting at random the pivot index (*i*) among the lattice points occupied by the arm; a random angle of rotation (+ 90°,− 90°,180°); and a random plane of rotation (*XZ* plane, *XY* plane, or *YZ* plane). The rotation $\theta_{i}$ is then applied to points above the pivot index. Rotations are accepted if arm self-avoidance is maintained and arm length measured along the *Z*-axis is maintained or increases. We use 350 rotation cycles for the autosomal arms and 300 for the X chromosome. The second phase of the Monte Carlo corresponds to step 3 (above) in which the right and left arms of chromosomes two and three are attached and folded at the centromere. To fold chromosome two and three at the centromere, we apply putative rotations identical to those in phase 1 with the exception that the pivot index (*i*) and centromere index (*j*) differ by no more than 100 lattice units. These rotations are accepted if chromosome self-avoidance is maintained and the distance between telomeres of the attached arms is maintained or decreases. We use 800 rotation cycles for chromosomes 2 and 3. In the third phase, putative rotations are made to reduce the area (or profile) of each curve in the *XY* plane. These putative rotations are identical to phase 1 but restricted to the *XY* plane of rotation. Rotations are accepted if chromosome self-avoidance is maintained and the occupied area in the *XY* plane is maintained or reduced. We use 6000 rotation cycles for each of the three chromosomes.

Step 4 details: The three chromosomes are repositioned such that the center of mass belonging to each curve occupies a point on the 13 × 13 × 5 unit lattice positioned at the center of the simulation box. Repositioning continues until the volume of the entire system is minimized while maintain self-avoidance of the three chromosomes. The entire lattice is then rescaled to match the diameter of each bead in simulation.

### Warm-up integration

The initial configurations generated with our Monte Carlo procedure (described above) have four key characteristics: (a) they are highly territorial, (b) they are free of entanglement, (c) they possess a fractal-like scaling exponent, and (d) they are arranged in a Rabl (polarized) configuration. However, these arrangements also embody the lattice used in their construction which diminishes their biological realism. We used a warm-up integration procedure to acclimate each structure to free space and establish confinement within the boundary representing the NE. The entire procedure is designed to simultaneously maintain the aforementioned key characteristics that are expected to deteriorate over time. First, a constraint sphere (representing the NE) is positioned at the center of the simulation box; its initial radius exceeds the radius of gyration of the chromosome configurations. The radius of the constraint sphere is incrementally reduced to its actual value during 2500 integration time steps. During these integration time steps, fractal-like structure of the chromosomes is preserved by increasing the Langevin friction coefficient and decreasing temperature: We set the temperature to 0 and the friction coefficient to 10^6^. An additional 1000 integrations steps are preformed after the constraint sphere assumes its final value. Once warm-up integration is complete, parameters of the Langevin equation (Eq. 5) are reset (see Table 2), and nuclear envelope attachments are turned on. A typical structure after warm-up integration is shown in Additional file 1 Figure S9 panel B.

### Checking robustness of the results to initial conditions

Each computational model comprises 1702 monomers confined within a boundary representing the NE. Simulation of the models consists of $\sim 10^{6}$ integration time steps preformed on Intel^®^ core i7 type CPUs: each simulation completed within several hours of real time. We checked that the conclusions in this study (see “Results” section) are robust to several key details of the computational model. Robustness to initial conditions was checked by changing the random seed value used during construction. Each random seed value generates a unique initial configuration prior to simulation. All model conclusions were reproduced using pairs of the wild-type model and control model stemming from different random seeds: a total of eight initial configurations were tested.

As discussed above, to calculate the fitting parameters, λ, used to rescale the simulation time, we use a plot of $\left\langle {\Delta \vec{r}^{2} (t)} \right\rangle$ for our *wild*-*type* model (with Chr–NE attachments) and determine its initial slope, 6*D*_sim_ (see above). We have checked that the value of the fitting parameter, *λ*, changes little when we use a plot of $\left\langle {\Delta \vec{r}^{2} (t)} \right\rangle$ generated with our *control* model (without the attachments).

We did not extensively investigate robustness of conclusions to the effects of model resolution; however, it has been noted in previous computational studies [10] and in theory [39] that polymer models are insensitive to coarse graining schemes above the Kuhn resolution.

### Chromosome territory index

**Fig. 9:**
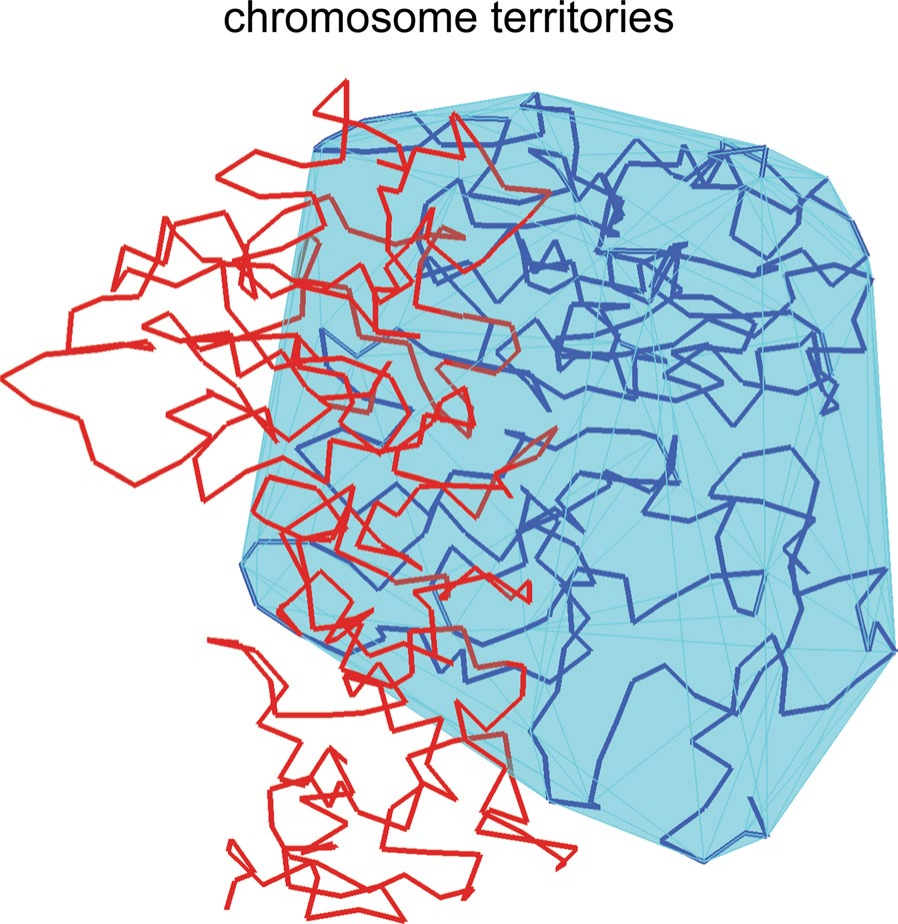
Territory index of a chromosome is defined as the percent of its beads found inside the chromosome’s own convex hull. Example: light blue chromosome inside its convex hull

Our definition of the territory had been used previously in similar contexts [28]. Briefly, we begin by calculating the convex hull for a single chromosome (blue chromosome in Fig. 3); this is the minimum volume that includes all the chromosome beads inside a convex polyhedron. In general, each convex hull contains its own chromosome and may also encompass some points belonging to other chromosomes (red chromosome in Fig. 9). A fully ‘‘territorial’’ chromosome is one whose convex hull does not contain beads from any other chromosomes while a less ‘‘territorial’’ chromosome is one whose convex hull contains some beads from other chromosome. We define the chromosome territory index as the fraction of beads inside a convex hull that belong to the chromosome used for its construction (Fig. 9).

### Chromosome entanglement

Theoretical studies suggest that fractal-like polymer configurations are non-entangled [13]. In previous studies, this lack of entanglement has been made quantitative with the concept of knot complexity which is computed by identifying knot invariant Alexander polynomials [13]. The absence of entanglement and knots within fractal-like configurations in turn facilitates chromosome folding, unfolding, and loop opening [13, 16]. Each of these properties makes fractal-like configurations biologically attractive; however, chromosomes tend to acquire knots as they transition from fractal-like configurations to equilibrium [13].

**Fig. 10:**
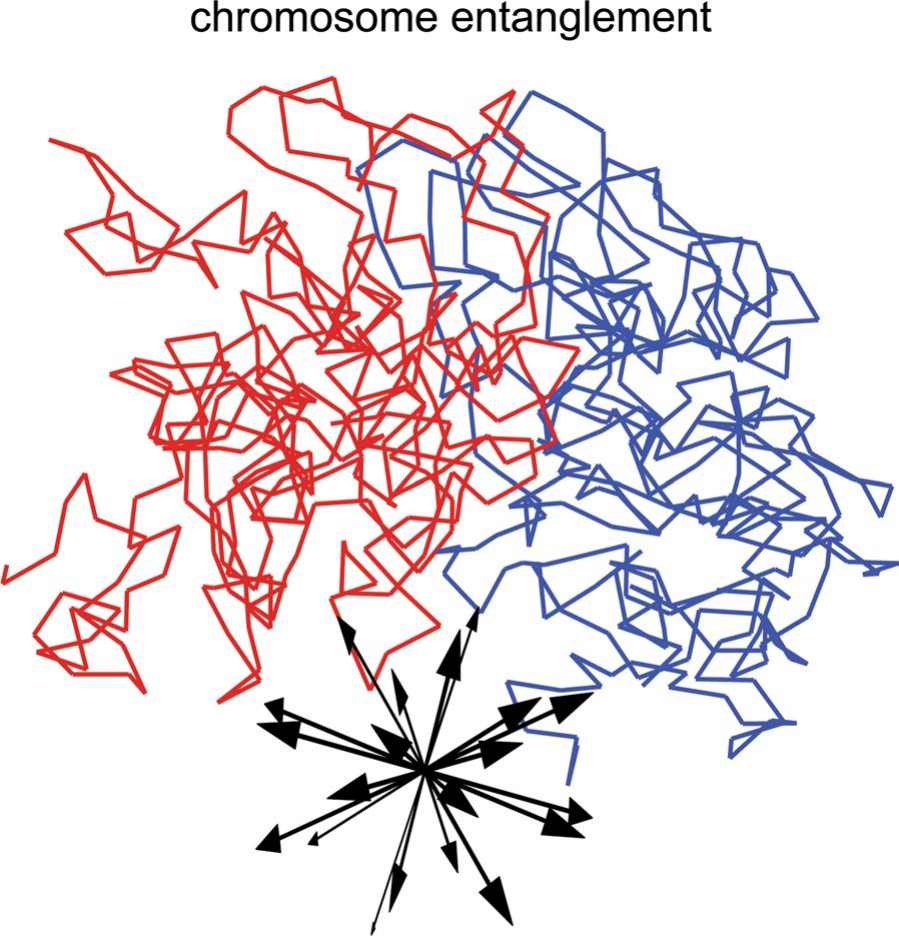
Spatial separation of chromosomes with respect to putative translations (inset) is used to quantify chromosome entanglement. The minimum number of crossings enumerated in 20 directions is used as a quantitative measure of chromosome entanglement

To quantify how entangled two chromosomes are in free space, we use a previously developed approach [28, 29]: We check whether two model chromosomes can be separated by putative translation in 3D space and enumerate the chain crossings along the direction of the applied translation (see Fig. 10). In general, the number of chain crossings in different directions will differ; therefore, we test 20 directions (Fig. 10) that uniformly cover the S2 space (spherical surface). From the 20 directions tested, the minimum number of crossings quantifies the entanglement of a pair of different chromosomes. Biologically speaking, this number is intended to represent how easily two chromosomes separate in free space.

## Additional file

**Additional file 1.** Description of data—companion model analysis

## Abbreviations

Chr–NE: chromosome–nuclear envelope
GFP: green fluorescent protein
Topo II: topoisomerase II
