# Supplementary material for: Chromosome–nuclear envelope attachments affect interphase chromosome territories and entanglement
Source: Epigenetics Chromatin. 2018 Jan 22;11:3. doi: 10.1186/s13072-018-0173-5 (PMC5776839; doi:10.1186/s13072-018-0173-5)
Supplement: Supplementary file 1 — Additional file 1. Description of data—companion model analysis [file 13072_2018_173_MOESM1_ESM.docx]

Supplemental Materials To:

Chromosome-nuclear envelope attachments affect interphase chromosome territories and entanglement.

Nicholas Allen Kinney^1^, Igor V. Sharakhov*^1,2,3^ and Alexey V. Onufriev*^1,4,5,6^

***Correspondence:** [igor@vt.edu](mailto:igor@vt.edu); [alexey@cs.vt.edu](mailto:alexey@cs.vt.edu)

**Author details:**

^1^ Genomics Bioinformatics and Computational Biology, Virginia Tech, Blacksburg, VA, 24061, USA

^2^ Department of Entomology, Virginia Tech, Blacksburg, VA, 24061, USA

^3^ Laboratory of Ecology, Genetics and Environmental Protection, Tomsk State University, Tomsk 634050, Russia

^4^ Department of Physics, Virginia Tech, Blacksburg, VA, 24060, USA

^5^ Department of Computer Science, Virginia Tech, Blacksburg, VA, 24061, USA

^6^ Center for Soft Matter and Biological Physics, Virginia Tech, Blacksburg, VA, 24061, USA

**Email addresses of the authors:**

Nicholas Allen Kinney ([nak3c@vt.edu](mailto:nak3c@vt.edu)), Igor V. Sharakhov ([igor@vt.edu](mailto:igor@vt.edu)), Alexey V. Onufriev ([alexey@cs.vt.edu](mailto:alexey@cs.vt.edu))

**Text S1 -** Robustness of the key results to the presence of ~50Mb of un-sequenced heterochromatin, included in our companion models (see “Methods” for details). The simulations based on these companion models reiterate the four main conclusion of this study: (a) Chr-NE attachments reinforce chromosome territories (figure S3); (b) Chr-NE attachments mitigate chromosome entanglement (figure S4); (c) Chr-NE attachments have little effect on the chromosome scaling exponent (figure S1); and (d) Chr-NE attachments do not affect the relaxation time of the Rabl chromosome configuration (figure S2).


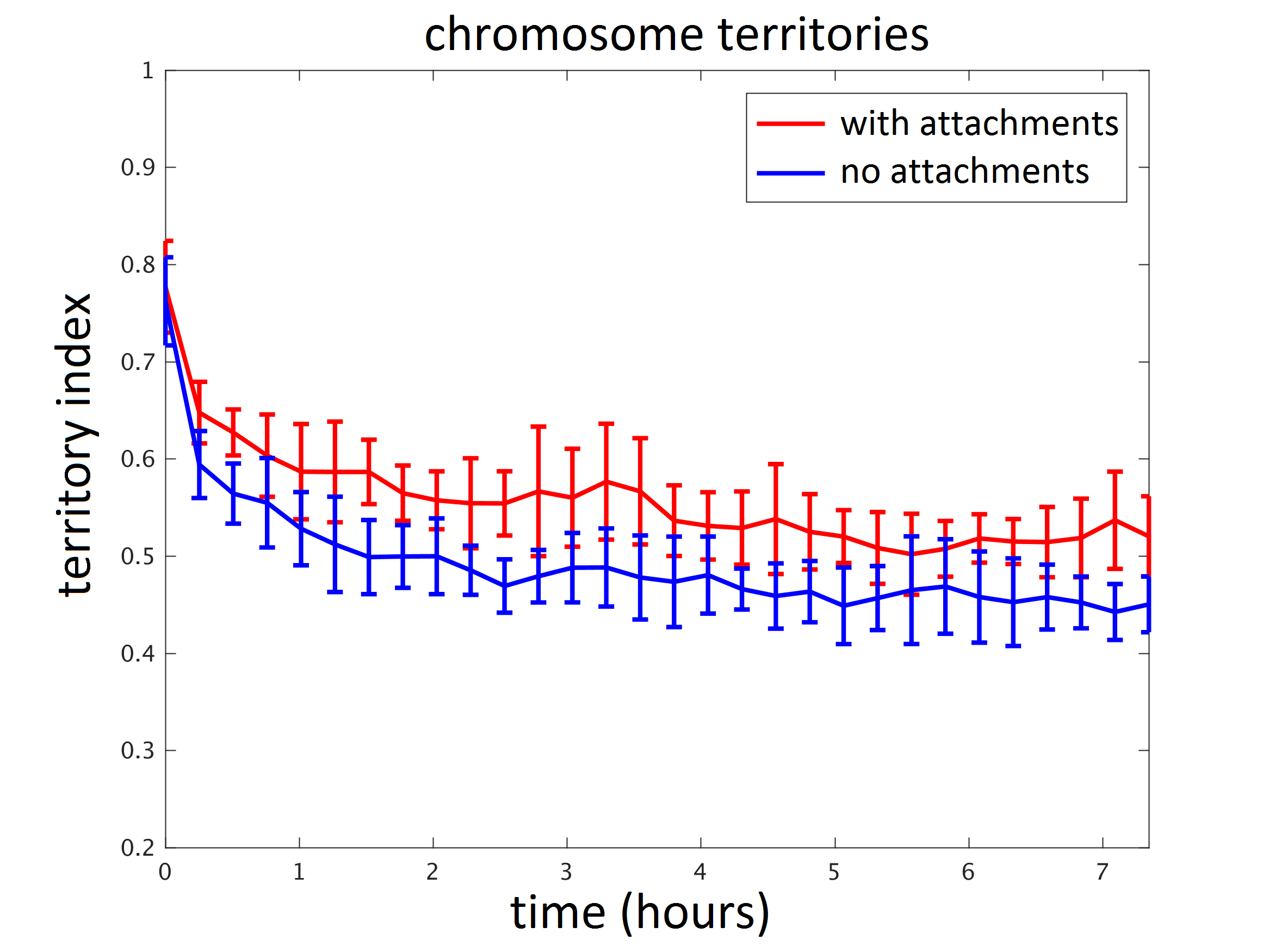


**Figure S3** - Effect of Chr-NE attachments on chromosome territories in companion models (heterochromatin present). Error bars represent 1 standard deviation calculated from n=8 simulation trajectories. Blue line – mean without attachments; red line – mean with attachments.


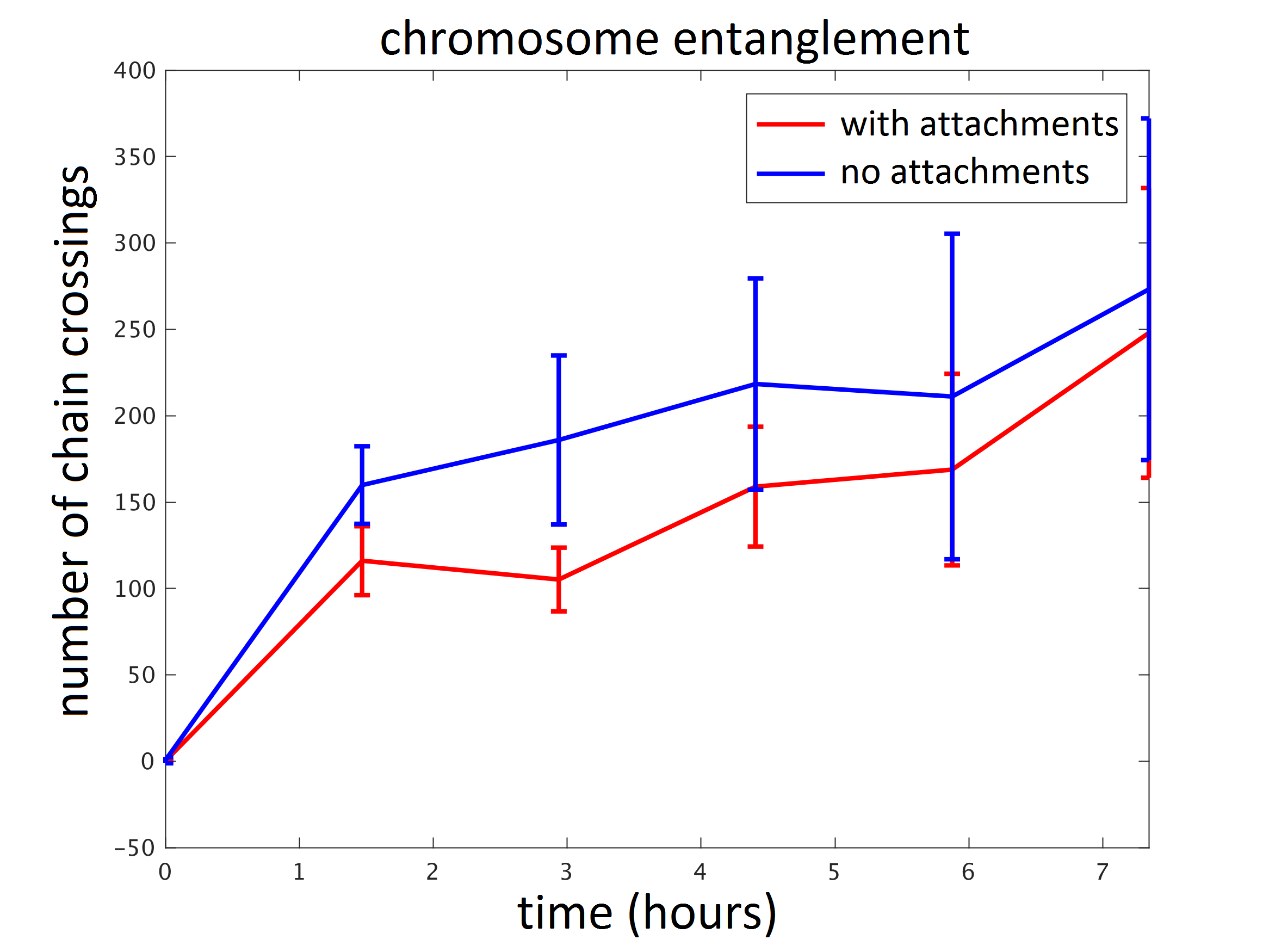


**Figure S4** - Effect of Chr-NE attachments on chromosome entanglement in companion models (heterochromatin present). Blue line – mean without attachments; red line – mean with attachments. Error bars represent 1 standard deviation calculated from n=8 simulation trajectories.


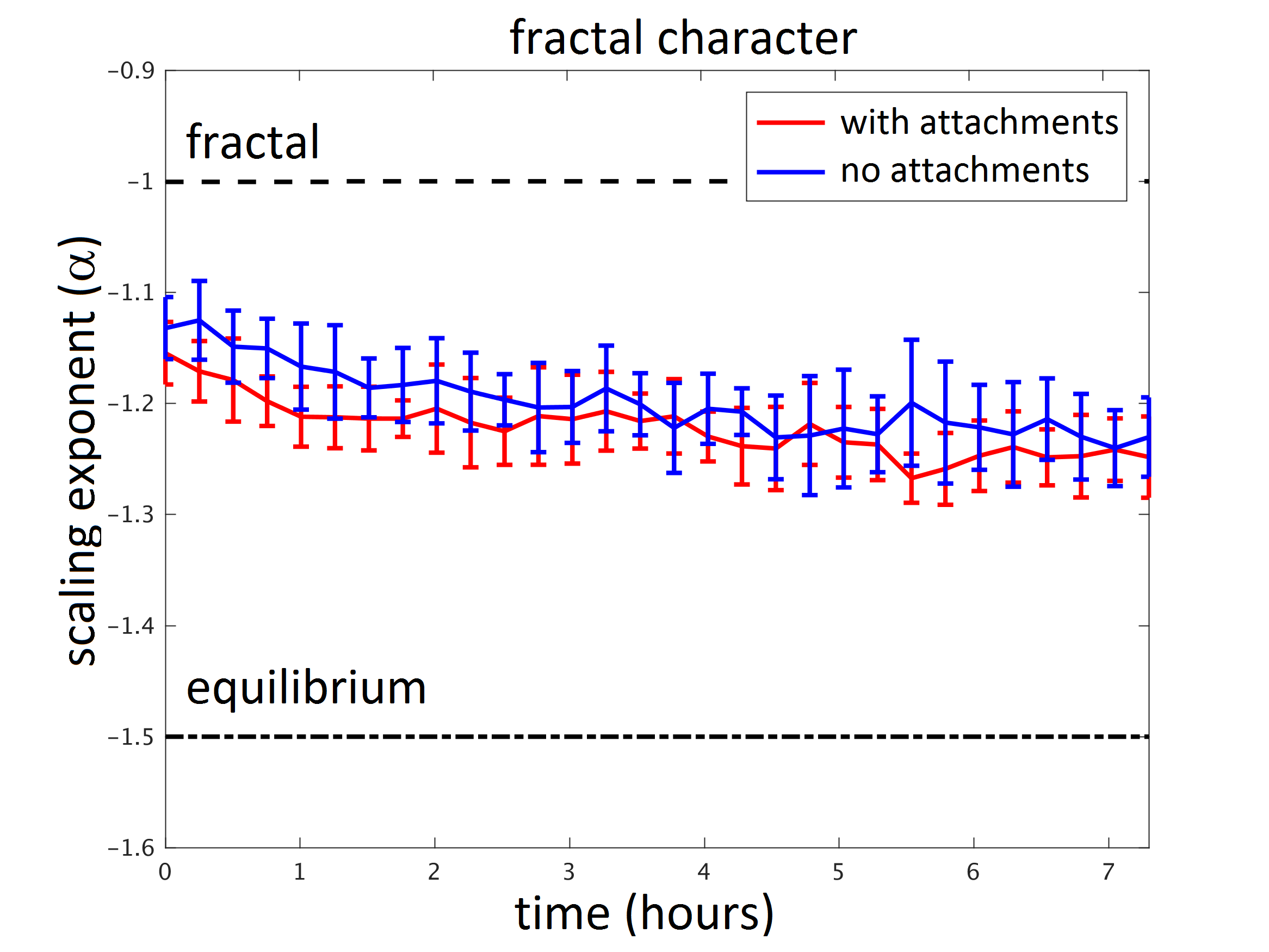


**Figure S1 -** Scaling of chromosome contacts in the presence of attachments and absence of attachments in companion models (heterochromatin present). Error bars represent 1 standard deviation calculated from n=8 simulation trajectories. Blue line – mean without Chr-NE attachments; red line – mean with Chr-NE attachments.


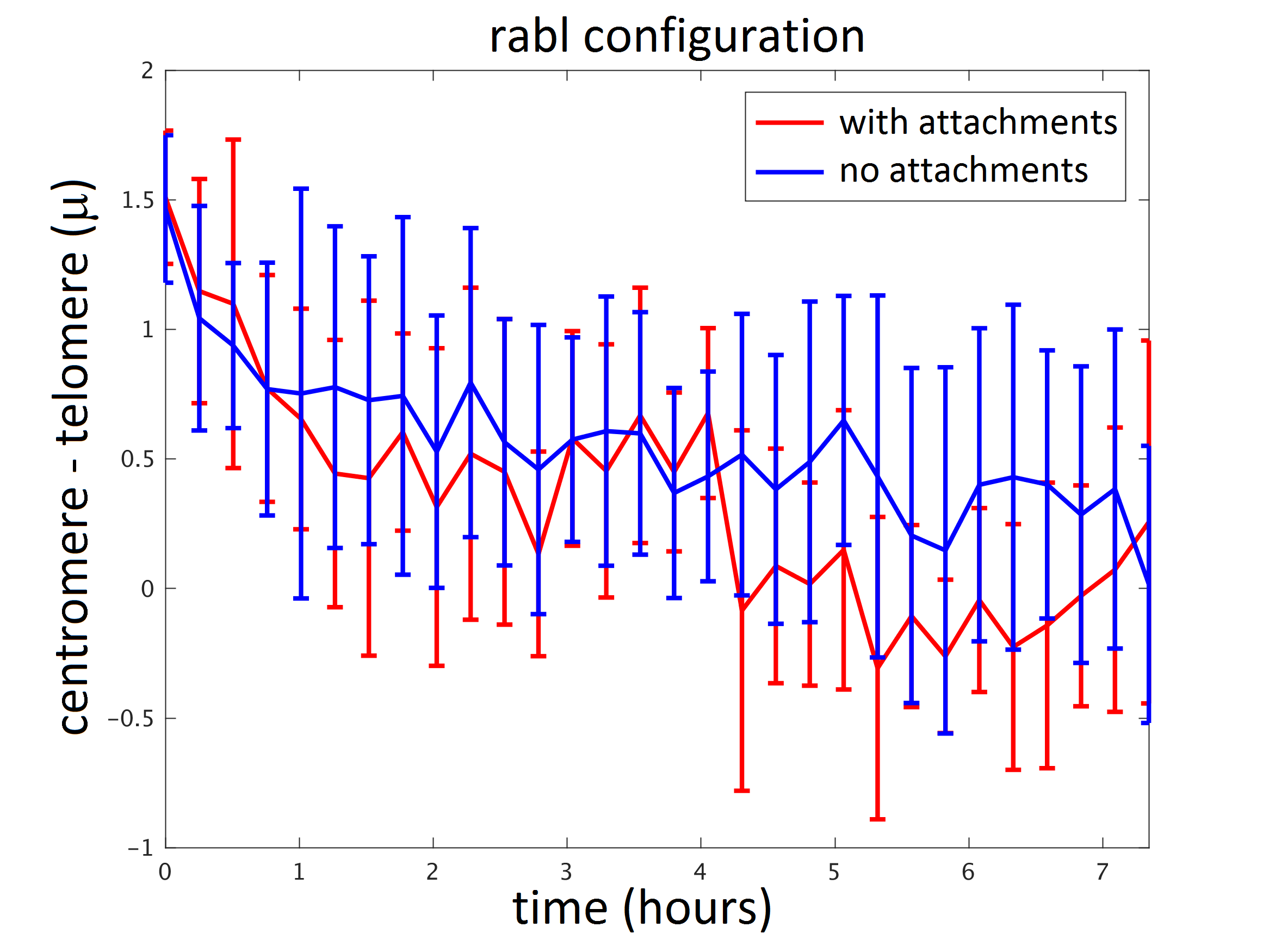


**Figure S2** - Effect of Chr-NE attachments on chromosome polarization (Rabl configuration) in companion models (heterochromatin present). Error bars represent 1 standard deviation calculated from n=8 simulation trajectories. Blue line – mean without attachments; red line – mean with attachments.

**Text S2 -** Robustness of the key results to the details of how Chr-NE attachments are modeled. We have considered a model with variable strength of Chr-NE interactions, see “Methods” . Simulations reiterate our 4 major conclusions: (a) Chr-NE attachments reinforce chromosome territories (figure S7); (b) Chr-NE attachments mitigate chromosome entanglement (figure S8); (c) Chr-NE attachments have little effect on the chromosome scaling exponent (figure S5); and (d) Chr-NE attachments do not affect the relaxation time of the Rabl chromosome configuration (figure S6).


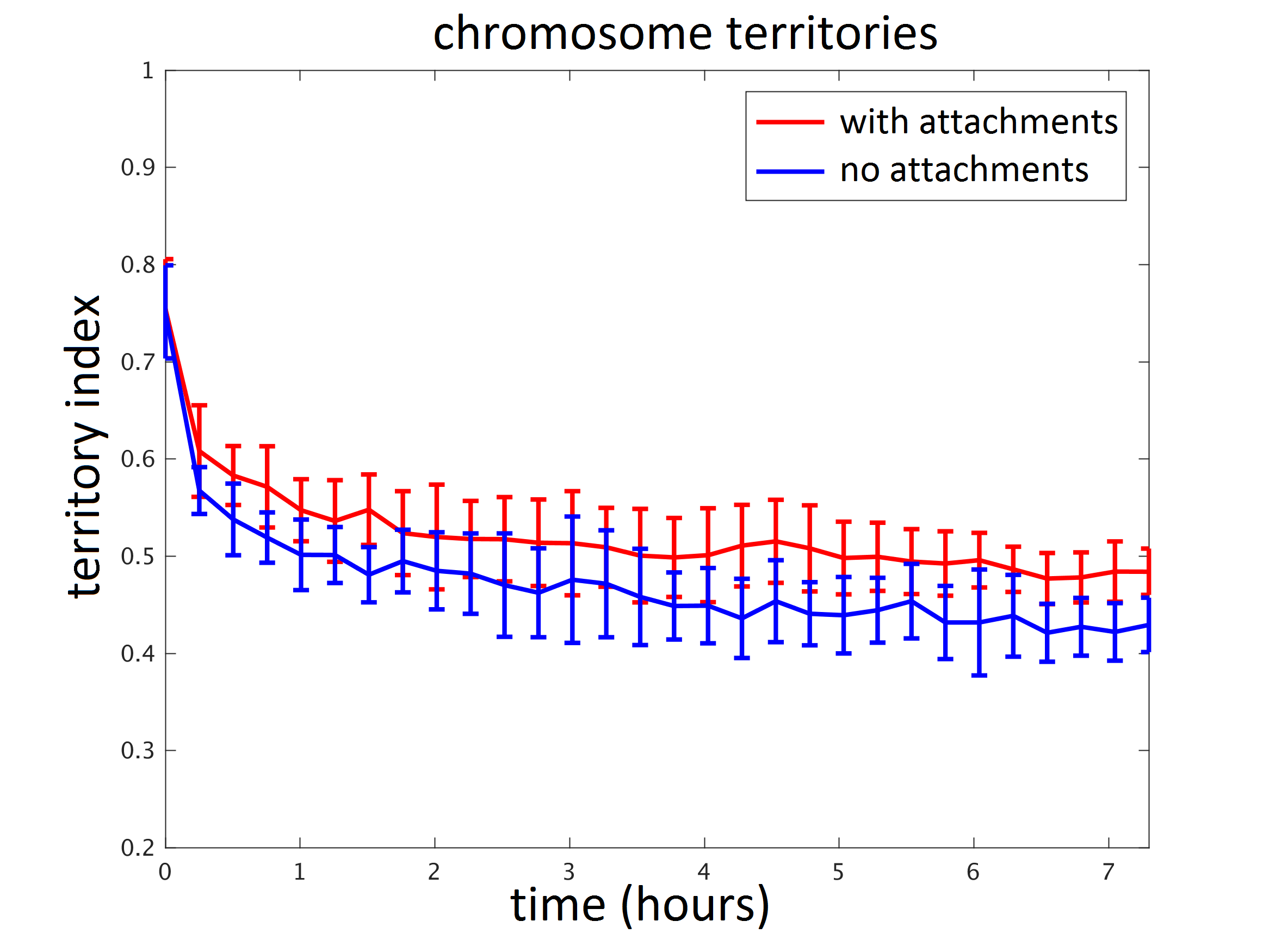


**Figure S7** - Effect of Chr-NE attachments on chromosome territories in models with variable strength of Chr-NE interactions. Error bars represent 1 standard deviation calculated from n=8 simulation trajectories. Blue line – mean without attachments; red line – mean with attachments.


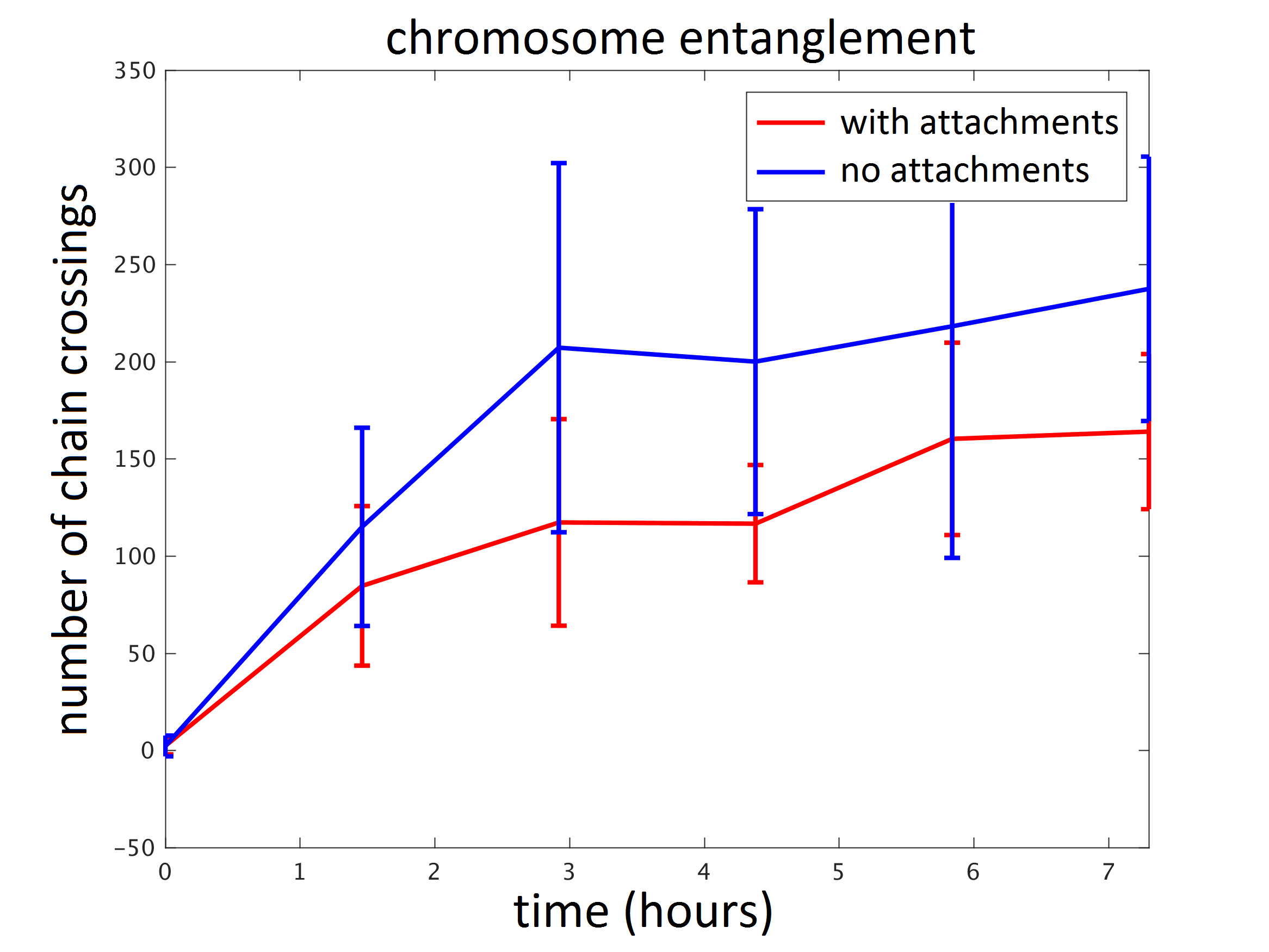


**Figure S8** - Effect of Chr-NE attachments on chromosome entanglement in models with variable strength of Chr-NE interactions. Blue line – mean with attachments; red line – mean with attachments. Error bars represent 1 standard deviation calculated from n=8 simulation trajectories.


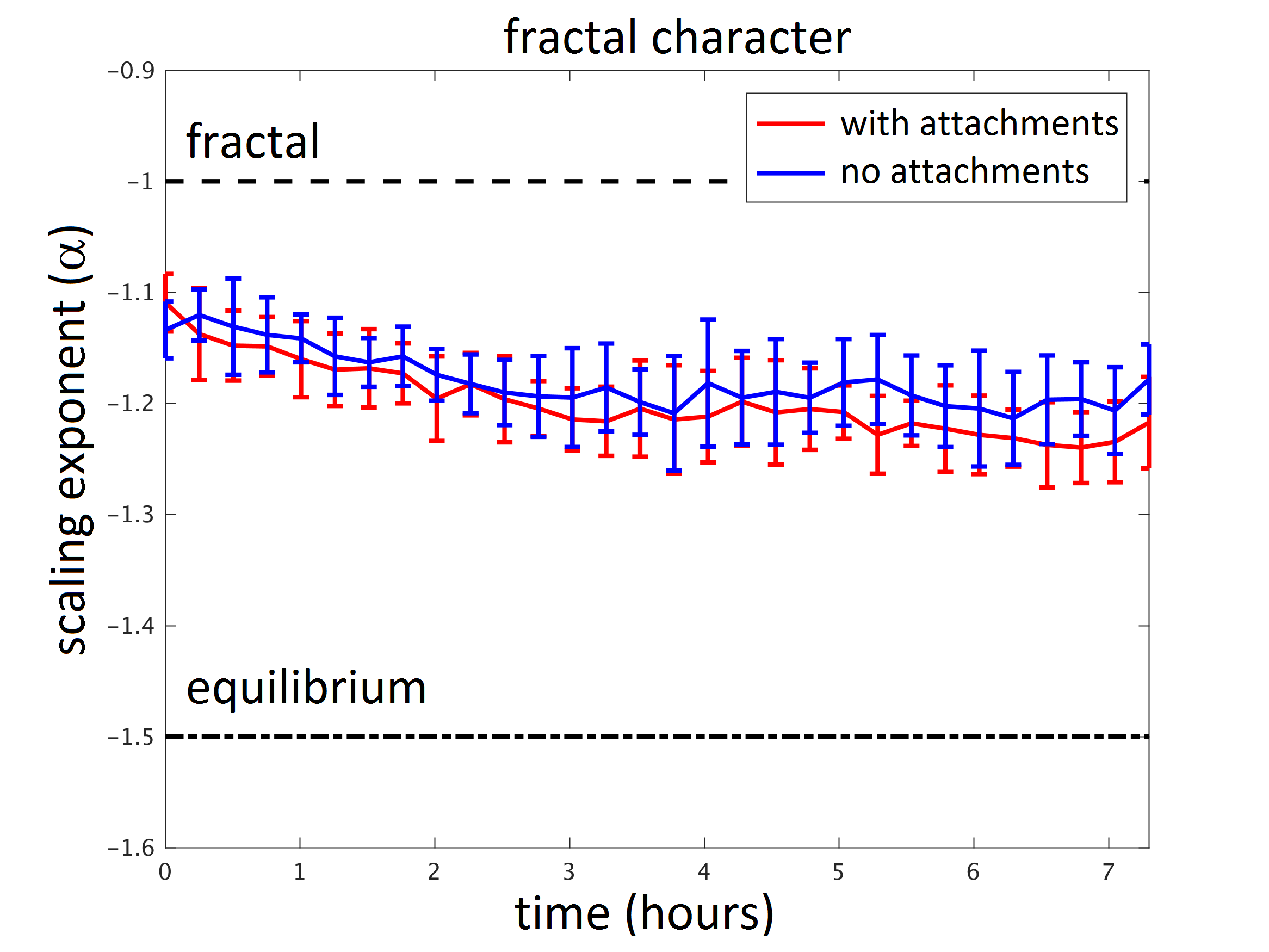


**Figure S5 -** Scaling of chromosome contacts in the presence and absence of attachments variable strength of Chr-NE interactions Error bars represent 1 standard deviation calculated from n=8 simulation trajectories. Blue line – mean without Chr-NE attachments; red line – mean with Chr-NE attachments.


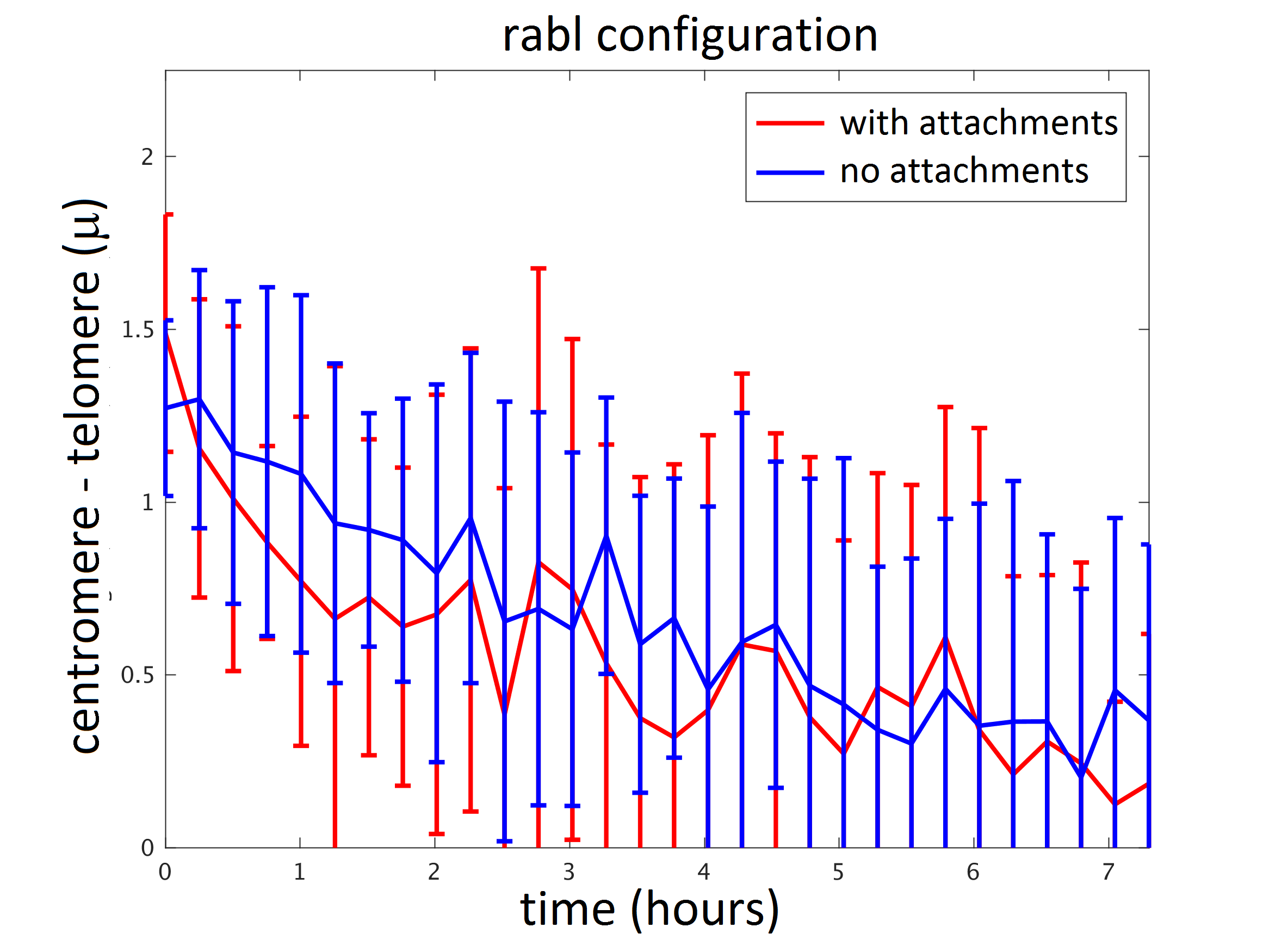


**Figure S6** - Effect of Chr-NE attachments on chromosome polarization (Rabl configuration) in models with variable strength of Chr-NE interactions. Error bars represent 1 standard deviation calculated from n=8 simulation trajectories. Blue line – mean without attachments; red line – mean with attachments.


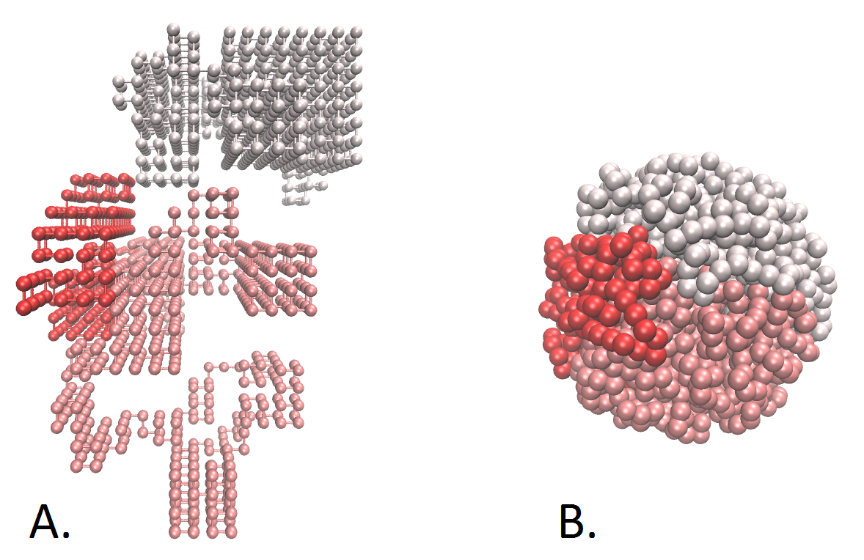


**Figure S9** - Procedure used to generate initial configurations. The snapshots above show a single model before (panel A) and after (panel B) warm up integration. The structure in panel A is generated using a Monte Carlo procedure that rearranges a precomputed Peano curve on a 3D-lattice. Then, warm up integration is applied to make the more realistic structure in panel B. See methods for details of the Peano curve rearrangements and warm up integration. All simulations begin from configurations typified by the structure in panel B. Chromosome in each initial configuration are territorial, compact (fractal-like), polarized (Rabl configuration), and free of entanglement. We find that combining lattice arrangements ( to arrive at panel A ) with subsequent free-space annealing ( to arrive at panel B ) computationally simplifies the task of enforcing these key features.
